# Memory-based quadratic interpolation optimization with reinforcement learning for robust PV parameter estimation

**DOI:** 10.1038/s41598-025-27626-1

**Published:** 2025-12-02

**Authors:** Mohamed Ghetas, Mohamed Issa

**Affiliations:** 1 Faculty of Computer Science and Engineering, Galala University, Suez, Egypt; 2 Computer and Systems Department, Faculty of Engineering, Zagazig University, Zagazig, Egypt; 3 Computer Science and Information Technology Programs, University for Science and Technology, Alexandria, Egypt

**Keywords:** Meta-heuristics, Quadratic interpolation optimization, Reinforcement learning, Solar cells, Parameter estimation, Energy science and technology, Engineering, Mathematics and computing

## Abstract

Solar cell parameter extraction is a critical yet challenging multimodal optimization problem, directly impacting the efficiency and modeling accuracy of photovoltaic (PV) systems. The Quadratic Interpolation Optimization (QIO) algorithm, while possessing strong exploitation capabilities, is prone to premature convergence and lacks a robust exploration mechanism. To overcome these limitations, this paper proposes a Memory-based Reinforcement Learning QIO (MRQIO) algorithm. MRQIO integrates a reinforcement learning agent to dynamically balance exploration and exploitation by adaptively adjusting search weights based on population diversity and fitness improvement. Furthermore, a memory-based mechanism leverages historical high-quality solutions to guide the quadratic interpolation process, enhancing global search capability and preventing stagnation in local optima. The proposed algorithm was rigorously validated on 13 benchmark functions and five distinct PV models: RTC France single-diode (SDM) and double-diode (DDM), STM6-40/36, STP6-120/36, and PWP 201. MRQIO demonstrated superior performance, achieving the lowest Root Mean Square Error (RMSE) values of 0.000986 (SDM), 0.000987 (DDM), 6.7794E-05 (STM6-40/36), 0.000014 (STP6-120/36), and 0.00243 (PWP 201). Comprehensive statistical tests, including the Wilcoxon rank-sum and Friedman tests, confirmed that MRQIO’s performance is significantly more accurate and robust than state-of-the-art metaheuristics, establishing it as a powerful tool for high-precision PV parameter estimation.

## Introduction

Recent studies predict that by 2050, 85% of the global population will live in cities. This significant urbanization will create a constant demand for specialized services^1^. Cities account for over 75% of global energy consumption and contribute 80% of greenhouse gas emissions^1,2^. To address the issue of pollution, adopting new environmentally friendly energy sources is essential^2^. Solar energy is one of the most profitable renewable sources currently being utilized to help meet the growing energy demand^3^. Solar energy is recognized for its quiet operation, ease of deployment, and non-polluting nature^4^.

Photovoltaic modules (PV), which are made up of solar cells (SC), are devices capable of converting solar energy into electricity. In other words, SC and PV modules must function effectively under varying weather conditions^5^, additionally, their maintenance should be cost-effective^6^. To accomplish these objectives, SC and PV modules must be carefully designed.

The design of SC involves the use of mathematical models to estimate the parameters that define the cell. These models are utilized to simulate the internal variables that govern the SC, aiming to represent the relationship between current and voltage (I-V). Two commonly used models are the single diode (SD) model and the double diode (DD) model. In^7^, the fundamental concepts of SD and DD modeling of PV solar cells are discussed.

The SD and DD models are electronic circuits that characterize the non-linearity of SC. Both models account for factors such as the photo-generated current, diode saturation current, series resistance, and diode ideality factor. The specific arrangement of these elements significantly influences the performance of the SC. The SD model is defined by five parameters, while the DD model uses seven parameters. Accurate estimation of these parameters is essential for achieving a precise balance in the current-voltage (I-V) relationship.

In recent years, various approaches have been introduced to optimally estimate the parameters of SC models. The methods can be categorized into three types: numerical techniques, analytical techniques, and soft-computing techniques^8,9^. Numerical techniques employ non-linear optimization methods, such as the Newton-Raphson technique^8^, and the conductivity method for estimating the parameters of SC^9^. The primary disadvantage of these techniques is their sensitivity to parameter initialization, which can result in being trapped in local minima, as the parameter estimation problem for SC is inherently multimodal^10^.

Numerous attempts have been made to estimate parameters using analytical methods, including the use of elementary functions and the Lambert W-function^11^. The primary issue with analytical methods is that they require more approximations due to the numerous parameters that need to be estimated, which results in increased time consumption^7^. Another drawback of the analytical method is that it requires additional coefficients, the values of which are not typically available in the datasheets^7^. As a result, analytical methods tend to be less accurate due to reliance on approximations and the absence of necessary data^12^.

Soft computing offers an alternative to address the shortcomings of numerical and analytical methods. Meta-heuristic algorithms (MA) seek optimal solutions based on a defined search strategy. MA is a high-level optimization strategy designed to efficiently find near-optimal solutions for complex and large-scale problems. Its main benefit when traditional exact algorithms become impractical due to time or computational constraints. Rather than guaranteeing the absolute best solution, MA aims to deliver good solutions within a reasonable timeframe, making them suitable for real-world applications.

The key advantages of metaheuristics lie in their flexibility, scalability, and global search ability. They can handle non-linear, non-convex, and multi-modal optimization problems where traditional methods may struggle. Furthermore, methodology is relatively easy to implement and adapt, offering a practical solution for researchers and practitioners dealing with complex decision-making problems. Various metaphors are employed in MA, such as the genetic algorithm (GA)^13^, based on the evolutionary theory. Meanwhile, physics-based algorithm methods like the sine-cosine algorithm (SCA)^14^ and the gravitational search algorithm (GSA)^15^. There is also another group of methods inspired by animals and insects, such as particle swarm optimization (PSO)^16^, artificial bee colony (ABC)^17^. MAs have been successfully applied in various fields, including the optimization of induction motor design^18^, sequence alignment^19,20^, suspension systems^21^ and PID controller tuning parameters^22,23^. MA explores multimodal search spaces using various operators to identify the optimal solution. When estimating solar cell parameters with MA, the root-mean-square error (RMSE) is employed as the objective function. The advantages of metaheuristics have motivated many recent research studies for optimizing the parameters of PV solar cells to enhance their efficiency. Parameter estimation for PV solar cells is a nonlinear, multimodal optimization problem, where the solution space has many local minima. If an algorithm focuses only on exploitation (local search), it may get trapped in a local minimum. If it relies too much on exploration (global search), it may wander without converging efficiently. The no-free-lunch (NFL) theorem asserts that no optimization technique can solve all problems effectively^24^.

In^25^, the GA is utilized to enhance the accuracy of the parameters estimated by the double diode model. PSO is applied to estimate the parameters of solar cells using both the SD and DD models^22^. Additionally, there has been a development of PSO that incorporates chaos theory to enhance exploration^26^. Furthermore, PSO has been utilized in solar fabrication with the support of neural networks^27^. Another approach employs simulated annealing (SA) to calculate the values of the SD and DD models^28^. According to the authors, the results of SA are superior to those of other approaches. Recently, the use of cat swarm optimization (CSO) has been proposed for identifying the optimal parameters of solar cells using both the SD and DD models^29^. In this context, different versions of the harmony search (HS) algorithm have been proposed to identify the unknown parameters of solar cells using the SD and DD models^30^. While such methods are efficient, they still face challenges related to accuracy. In practical applications like SC model identification, achieving accurate outputs is essential to minimize the costs associated with energy systems^31^. In light of this, an improved cuckoo search algorithm (ImCSA) has been proposed in^32^ to improve the estimation of parameters for PV cells using the diode models. Other related works are the bird-mating optimizer^33^ and pattern search (PS)^34^.

In^35^, a novel hybrid flower grey differential (HFGD) algorithm was proposed, which combines the flower pollination algorithm, grey wolf optimizer, and differential evolution, to accurately estimate the unknown parameters of PV systems. By integrating Newton–Raphson refinement and testing on multiple PV models, the method demonstrates superior precision, convergence, and robustness compared to existing evolutionary algorithms. Hippopotamus optimizer was also used for accurate parameter identification in PV models, including single-diode, double-diode, and the Sandia PV array performance model^36^. The optimizer was evaluated on eight commercial PV units of different technologies, demonstrating its ability to minimize root mean square error and closely match modeled and actual I–V and P–V characteristics. Besides, an improved marine predators algorithm (MPA) for parameter estimation in PV systems, aiming to overcome the limitations of existing optimization techniques, such as high computational cost and susceptibility to local optima^37^. The improved MPA incorporated a population improvement strategy, combining adaptive mutation for high-quality solutions and guided updates for low-quality solutions to enhance overall performance. Experimental evaluations across multiple PV models demonstrate that the improved MPA achieved superior accuracy and strong correlation with measured I–V data.

A hybrid optimization approach called PIFN, which integrated the Prairie Dog Optimization, INFO, Fission Fusion, and Naked Mole-Rat algorithms to improve exploration, exploitation, and local optima avoidance in PV parameter estimation^38^. The framework incorporated five new mutation operators/inertia weights and introduced a stagnation phase, making the algorithm self-adaptive across varying population sizes and dimensions. Extensive testing on benchmark functions and photovoltaic modules, supported by statistical analyses, demonstrated that PIFN consistently delivers superior accuracy, achieving the lowest RMSE values compared to other metaheuristic methods. An enhanced Artificial Rabbit Optimization algorithm that integrates swarm-elite learning, Lévy flight, and individual mutation to tackle the complex problem of parameter extraction in PV models^39^. This method enhances global exploration by incorporating elite information and Lévy jumps, and boosting population diversity with mutation-based perturbations, thereby achieving more reliable optimization outcomes. Comparative evaluations across benchmark functions and five PV models confirmed their superior accuracy, faster convergence, and consistently low RMSE values compared to state-of-the-art metaheuristics and classical methods.

The Pelican Optimization Algorithm (POA) is a robust technique for solving the nonlinear parameter extraction problem in PV models^40^. Using the Single Diode four benchmark datasets, the method integrated Newton–Raphson refinement to enhance accuracy and convergence. Experimental and statistical results demonstrated that POA consistently achieves lower RMSE values and higher robustness compared to other metaheuristic algorithms, confirming its effectiveness in optimizing the performance of PV systems. Another study investigated parameter estimation in multi-junction solar cells (MJSCs) using three mathematical models Single Diode Model, the Double Diode Model, and the Triple Diode Model to improve accuracy in energy output prediction and fault detection^41^. A hybrid approach combining the Newton–Raphson Method with metaheuristic optimization algorithms was applied to enhance convergence speed, reduce computational costs, and ensure robust parameter extraction. Comparative results highlight the Artificial Hummingbird Algorithm (AHA) as the best performer for SDM and DDM, while the War Strategy Algorithm (WSA) achieves the lowest RMSE for TDM, demonstrating the effectiveness of tailored algorithm-model combinations in MJSC optimization.

A comparative study of 11 metaheuristic algorithms for solar cell parameter identification, emphasizing the trade-off between computational efficiency and accuracy^42^. The Pattern Search algorithm consistently outperformed others in terms of stability, precision, and convergence speed. Statistical comparisons confirm its reliability, highlighting its potential as a robust tool for enhancing solar energy modeling and maximum power point retention. Newton-Raphson-Based Optimizer (NRBO), a novel metaheuristic designed to improve the accuracy and efficiency of PV parameter extraction^43^. NRBO achieves a better balance between exploration and exploitation while avoiding local optima. Experimental validation on the RTC France PV cell shows that NRBO outperforms established algorithms such as GWO, DO, WOA, and AOS, delivering lower error values, faster convergence, and higher reliability in real-world PV modeling applications.

The problem statement of this study is the optimization of the parameter estimation of the PV solar cell using the Quadratic Interpolation Optimization (QIO) algorithm^44^ according to NFL theory. QIO^44^ was introduced to tackle numerical optimization and engineering challenges. The primary inspiration for QIO comes from mathematics, particularly the recently developed generalized quadratic interpolation (GQI) method. This method addresses the limitations of the conventional quadratic interpolation approach, enhancing its ability to identify the minimizer of the quadratic function formed by any three points. The QIO employs the GQI method as an effective search mechanism for addressing a variety of optimization problems. This search mechanism incorporates exploration and exploitation strategies, where the minimizer identified by the GQI method helps the QIO algorithm to explore promising regions in uncharted areas while also exploiting optimal solutions in known promising regions.

The exploitation capability of the QIO is one of its strongest features, particularly due to its focus on locally refining solutions. Since QIO models the objective function locally as a quadratic function, it allows it to effectively exploit the search space around promising regions to refine solutions. This motivates the use of QIO for PV parameters estimation since efficient exploitation in balance with the exploration phase is critical for accurately estimating PV parameters due to the complex, non-linear nature of the optimization problem. Besides, QIO has low noise sensitivity in local refinement since it does not depend on derivatives but uses interpolation; it is robust against minor noise in function evaluations, making it suitable for exploitation in slightly noisy environments.

The QIO algorithm has been used to enhance many engineering problems with enhanced versions. Such as in^45^ presents a deep learning model for diagnosing faults in hydro-turbines that utilize chaotic quadratic interpolation optimization (CQIO). Chaotic mapping enhances the initial population of the QIO algorithm by incorporating randomness and diversity, thereby improving the algorithm’s performance and stability. The CQIO was employed to determine the optimal hyperparameter combinations for CNN-LSTM models, which help enhance the model’s stability and minimize the consumption of computational resources. In^46^ presents a novel approach to load frequency control (LFC) by proposing a filtered PID (PID-F) controller optimized using a hybrid simulated annealing-based quadratic interpolation optimizer (hSA-QIO). The hSA-QIO effectively merges the local search capabilities of simulated annealing with the global optimization strengths of the QIO, resulting in a robust and efficient solution for LFC challenges. The main contributions of this study include the development and application of the hSA-QIO, which significantly improves the performance of the PID-F controller.

In^47^, a novel optimization method was presented, known as the Quadratic Interpolation-enhanced Artificial Gorilla Troops Optimizer (QIGTO), specifically developed to tackle these challenges. Besides, a flux sliding mode observer (QIO-FSMO) method that utilizes quadratic interpolation optimization for senseless control of permanent magnet synchronous motors (PMSM) was proposed^48^. QIO is employed to optimize the observer gain of FSMO in real time, enhancing the system’s ability to suppress chattering. Simulation results demonstrate that the QIO-FSMO method significantly reduces chattering in the senseless control system of permanent magnet synchronous motors (PMSM) and exhibits strong robustness. QIO was used in the Flood forecasting domain, where it is considered a critical non-engineering measure, with its accuracy being essential for effective flood control and regulation^49^. The conceptual rainfall–runoff model (CRR) is commonly used for flood forecasting. However, a significant challenge in using CRR models in hydrology is their calibration, as these models typically involve many parameters.

However, applying QIO for these research studies, the main limitation of QIO is prone to premature convergence and often gets stuck in local optima. In addition, it lacks an exploration mechanism, which limits its ability to explore new promising search spaces. To address these challenges to be adaptable to PV cell parameter estimation, an enhanced version of QIO is proposed based on reinforcement learning and memory-based mechanism.

The main contributions of this work are as follows:

1. A novel enhanced QIO algorithm is developed by integrating reinforcement learning for adaptive balancing of exploration and exploitation, overcoming the inherent limitations of standard QIO.
2. A memory-based mechanism is introduced to prevent premature convergence, increase diversity, and improve global search capability.
3. Robust exploitation ability is preserved by leveraging QIO’s interpolation-based refinement, ensuring accurate local search even under noisy conditions.
4. Comprehensive benchmarking of the proposed algorithm is performed against state-of-the-art metaheuristics on standard test functions to validate its stability, convergence, and robustness.
5. Application to PV parameter estimation is carried out on multiple PV models, demonstrating superior accuracy, convergence speed, and robustness compared to existing algorithms in the literature.
6. Statistical validation is provided to prove the significance of the proposed algorithm over its counterparts.

The organization of this paper considers the following sections: Sect. 2 presents the diode models utilized for solar cells and the formulation of the optimization problem. In Sect. 3, the preliminaries of QIO are presented. Section 4 describes the enhancement of QIO. Section 5 shows the evaluation metrics and datasets. Section 6 shows the experimental methodology and presents the results. Meanwhile, Sect. 7 includes some conclusions and future work.

## Formulation of PV parameter estimation problem

This section presents the mathematical formulations and structural representations of both the single diode (SD) and double diode (DD) models, highlighting their components, governing equations, and the parameters that must be estimated for accurate simulation.

### Single diode (SD) circuit model

**Fig. 1:**
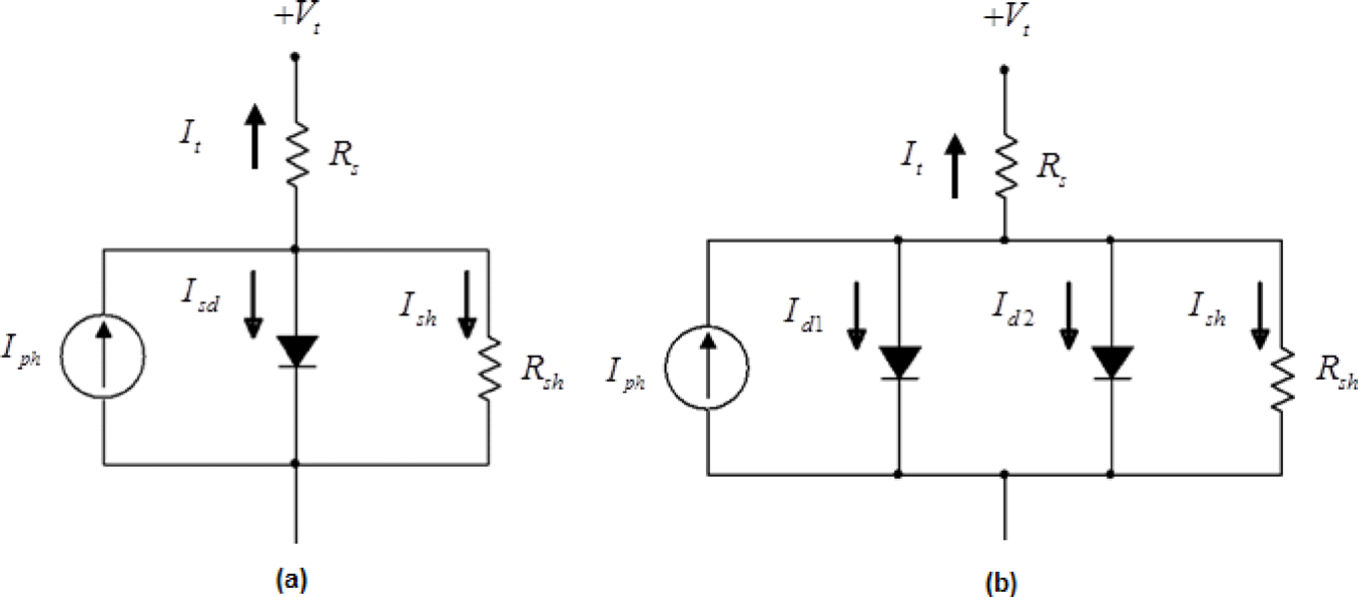
The Representation circuit of (**a**) SD (**b**) DD models.

This model includes a single diode used to shunt the photogenerated current source, as illustrated in Fig. 1 (a), where the diode represents the rectifier in the circuit. Generally, the SD model requires the estimation of five parameters, as their configuration significantly impacts the model’s output.

In general, the short-circuit current (${I_t}$) is calculated using the following equation:

1 $$\:{I}_{t}=\:{I}_{ph}-{I}_{d}-\:{I}_{sh}$$

where ${I_{sh}}$, ${I_t}$,${I_d}$ and ${I_{ph}}$ the currents through the shunt resistor, the terminal, the diode, and the photogenerated current are considered, respectively. Based on the equivalent Shockley diode equation, the internal parameters of the diode can be adjusted to enhance performance. Accordingly, Eq. 1 can be rewritten as:

2 $$\:{I}_{t}=\:{I}_{ph}-\:{I}_{sd}\:\left[\text{exp}\left(\frac{q\:\left({V}_{t}+\:{R}_{s}\:.\:{I}_{t}\right)}{n.k.T}\right)-1\right]-\:\frac{{V}_{t}+\:{R}_{s}\:.\:{I}_{t}}{{R}_{sh}}$$

where ${V_t}$, ${I_{sd}}$, ${R_{sh}}$and ${R_s}$ parameters correspond to the terminal voltage, the diode saturation current, the shunt resistance, and the series resistance, respectively. The variable represents the non-physical ideality factor. Also, $q=1.602 \times {10^{ - 19}}$ C (coulombs) represents the magnitude of an electron’s charge. Meanwhile, $k=1.380 \times {10^{ - 23}}$ $({J \mathord{\left/ {\vphantom {J {^\circ K}}} \right. \kern-0pt} {^\circ K}})$ and *T* are the Boltzmann constant and the cell temperature (*ºK*), respectively.

### Double diode (DD) circuit model

This section introduces the description of the DD model, where one diode serves as the rectifier while the second diode accounts for the current and other non-idealities of the solar cell. The DD model is illustrated in Fig. 1(b). Based on Fig. 1(b), Eq. 1 can be rewritten as follows:

3 $$\:{I}_{t}=\:{I}_{ph}-\:{I}_{d1}-{I}_{d2}-\:{I}_{sh}$$

where ${I_{d1}}$ and ${I_{d2}}$ are currents of the first and second diodes are represented, respectively. The Shockley equivalence is applied to modify the internal configuration of the diodes provided in Eq. 3, resulting in the following form:

4 $$\:{I}_{t}=\:{I}_{ph}-\:{I}_{sd1}\:\left[\text{exp}\left(\frac{q\:\left({V}_{t}+\:{R}_{s}\:.\:{I}_{t}\right)}{{n}_{1}.k.T}\right)-1\right]-{I}_{sd2}\:\left[\text{exp}\left(\frac{q\:\left({V}_{t}+\:{R}_{s}\:.\:{I}_{t}\right)}{{n}_{2}.k.T}\right)-1\right]-\:\frac{{V}_{t}+\:{R}_{s}\:.\:{I}_{t}}{{R}_{sh}}$$

where${I_{sd1}}$ and ${I_{sd1}}$ are diffusion and saturation currents for the diodes $\:{d}_{1}$ and $\:{d}_{2}\:$are represented, respectively. The variables $\:{n}_{1}$and $\:{n}_{2}\:$are the diffusion and recombination diode ideality factors, respectively. From Eq. 4, the Double Diode (DD) circuit includes seven undefined parameters. (i.e., ${R_s},{\text{ }}{R_{sh}},{\text{ }}{I_{ph}},{\text{ }}{I_{sd1}},{\text{ }}{I_{sd2}},{\text{ }}{n_1}{\text{ and }}{n_2}.$) needed to be estimated.

## Quadratic interpolation optimization (QIO) algorithm

### Preliminary concept

The Quadratic Interpolation Optimization algorithm tackles the optimization problem by finding a quadratic interpolation polynomial function $\:L\left(x\right)$that approximates the optimization problem $\:f\left(x\right)$. Finding the minimizer or maximizer of the function $\:L\left(x\right)$ approximates the minimizer or maximizer of $\:f\left(x\right)$. The function $\:L\left(x\right)$ can be expressed as:

5 $$\:L\left(x\right)=a{x}^{2}+bx+\:c \:a,b,c\in\:R\: {\rm and\: a\: \leqslant\: b}$$

If three points $\:{(x}_{1},{y}_{1}),$ $\:{(x}_{2},{y}_{2})$ and $\:{(x}_{3},{y}_{3})$ on the curve of $\:L\left(x\right)$ are known and within the interval $\:[a,b]$ then the minimizer $\:{x}^{\text{*}}$ can be determined by taking the derivative and setting it to zero as:

6 $$\:\frac{dL}{dx}=2ax+b$$

Solving for $\:{x}^{\text{*}}$ give that

7 $$\:{x}^{*}=-\frac{b}{2a}$$

The coefficients $\:a$ and $\:b$ can be identified using the three known points. The selection of the three points is considered a significant challenge, as an improper selection can lead to finding a minimizer rather than a maximizer and vice versa. To address this challenge, the Generalized Quadratic Interpolation (GQI) technique is used. GQI searches for the appropriate function $\:L\left(x\right)$ by finding various possible cases for selecting the three points. Once GQI identifies the optimal three points, the minimizer $\:{x}^{\text{*}}$ can be calculated as follows

8 $$\:{x}^{*}=\frac{\left({x}_{2}^{2}-{x}_{3}^{2}\right)\:{y}_{1}+\left({x}_{3}^{2}-{x}_{1}^{2}\right){y}_{2}+\left({x}_{1}^{2}-{x}_{2}^{2}\right)\:{y}_{3}}{2[\left({x}_{2}-{x}_{3}\right){y}_{1}+\:\left({x}_{3}-{x}_{1}\right){y}_{2}+\:\left({x}_{1}-{x}_{2}\right){y}_{3}]}$$

### Exploration strategy

Exploration strategy in optimization algorithms refers to the capability of the algorithm to discover candidate solutions within the search space and avoid trapping in local minima. The GQI is used in the QIQ algorithm to explore the search space. The GQI identifies the promising search space by randomly choosing two individuals $\:{x}_{r1}\left(t\right),$ $\:{x}_{r2}\left(t\right)$ from the current population and joining them with the current individual to identify the minimizer $\:{x}^{\text{*}}\:$of the optimization function $\:L\left(x\right)$. Meanwhile, the new candidate solution is generated from the minimizer $\:{x}^{\text{*}}$ and a third individual $\:{x}_{r3}\left(t\right)$ randomly chosen from the current population. The following equations demonstrate the calculation of the minimizer $\:{x}^{\text{*}}$ as well as the new candidate solution $\:{x}_{new}$

9 $$\:{x}_{new}\left(t+1\right)=\:{x}^{\text{*}}\left(t\right)+\:{w}_{1}\:\left({x}_{r3}\left(t\right)-{x}^{\text{*}}\left(t\right)\right)+round\left(0.025+0.5{r}_{1}\right).\:\:log\frac{r2}{r3}\:$$

10 $$\:{x}^{\text{*}}\left(t\right)=GOI\:(x\left(t\right),\:{x}_{r1}\left(t\right), \:{x}_{r2}\left(t\right))$$

Where $\:{w}_{1}$ is the exploration weight and is calculated based on the current iteration as indicated in^35^, and $\:{r}_{1}$, $\:{r}_{3}$, and $\:{r}_{3}$ are random numbers in (0,1).

### Exploitation strategy

This strategy uses local search to refine the best solution found such far and converges to a global solution. The GOI method randomly chose two individuals $\:{x}_{r1}\left(t\right)\:$and $\:{x}_{r2}\left(t\right)\:$from the current population and joining them with the best solution $\:{x}_{best}\left(t\right)$ to formulate the minizer $\:{x}^{\text{*}}\left(t\right)$. Then QIQ promises to find a new individual around to the minimizer x* as follows

11 $$\:{x}_{new}\left(t+1\right)=\:{x}^{\text{*}}\left(t\right)+\:{w}_{2}.\:\:\left({x}_{best}\left(t\right)-round\:(1+r\right))+\frac{u-l}{{u}^{d}-{l}^{d}}.\:{x}^{d}(t)\:\:$$

 

12 $$\:{x}^{\text{*}}\left(t\right)=GOI\:({x}_{best}\left(t\right),\:{x}_{r1}\left(t\right),\; \:{x}_{r2}\left(t\right))$$

Where $\:{w}_{2}$ is an exploitation weight that adaptively changes depending on the current iteration, $\:{u}^{d}$and $\:{l}^{d}$ are the upper and the lower boundaries at the d-dimension.

## The proposed MRQIO

The GOI strategy discovers the promise search region by selecting three points using random sampling through the exploration stage. Conversely, in the exploitation strategy, GOI leverages the interpolated quadratic function to calculate the minimizer x* based on existing knowledge. However, Reinforcement Learning (RL) can be trained to observe the optimization progress, such as the fitness function, and the diversity of the population. Accordingly, the RL can learn the optimal policy to adaptively change the exploration weight $\:{w}_{1}$ as well as exploitation weigh $\:{w}_{2}$ that controls the selection of GOI’s point selection.

This adaptation enables the algorithm to explore undiscovered search areas, which can lead to avoiding trapping in local minima and refining the obtained solution. Consequently, the convergence is improved, especially in problems with a large and complex search space.

The RL defines the states using two quantities, which are fitness improvement ($\:\varDelta\:f$) and the population diversity $\:\left(D\right)$. The improvement on best fitness can be discretized into four categories as high improvement ($\:{\varDelta\:f}_{\text{\%}}$ >20), low improvement ($\:1\:\le\:{\varDelta\:f}_{\text{\%}}<10$), moderate improvement ($\:10\text{\%}\le\:\:{\varDelta\:f}_{\text{\%}}<20$), and no improvement $\:{\varDelta\:f}_{\text{\%}}\le\:0$).

The fitness improvement can be calculated as the difference between the best fitness at the current iteration $\:\left(t\right)\:$and the previous iteration ($\:t-1)$ as shown in Eq. 13.

13 $$\:{\varDelta\:f}_{\%}=\:\frac{f\left({x}_{b}^{t-1}\right)-f\left({x}_{b}^{t}\right)}{f\left({x}_{b}^{t-1}\right)}\times\:100$$

Where $\:f\left({x}_{b}^{t-1}\right)$, and $\:f\left({x}_{b}^{t}\right)$ are the best fitness value in the previous and current iterations, respectively.

The population diversity (D) measures how the agent spreads across the search space to find candidate solutions. The population diversity is discretized into three levels:

- Low diversity ($$\:D\:<0.1$$).
- Medium diversity ($$\:0.1\:\le\:D\:<0.5$$).
- High diversity ($$\:D\:\ge\:0.5$$).

The population diversity can be calculated as shown in Eq. 14.

14 $$\:D=\:\frac{1}{n}\sum\:_{i=1}^{n}\left|\left|{x}_{i}-{x}^{-}\right|\right|\:,\:{x}^{-}=\frac{1}{n}\sum\:_{i=1}^{n}{x}_{i}$$

where $\:{x}_{i}$ represents the mean value of the individual, and $\:n$ is the number of populations. According to the combination of the fitness improvement ($\:\varDelta\:f$) and population diversity (D). MRQIO has 12 states and according to the current state of MRQIO, the RL agent adjusts the values $\:{w}_{1}$, and $\:{w}_{2}$ as follows

15 $$\:{w}_{1}\:=\:{w}_{1}+\:\varDelta\:w$$

16 $$\:{w}_{2}\:=\:{w}_{2}+\:\varDelta\:w$$

where $\:\varDelta\:w\:\in\:\left[\text{0,1}\right]$ and determine how much the parameters $\:{w}_{1}$, and $\:{w}_{2}$ are adjusted during each iteration. Therefore, the agent can increase/decrease $\:{w}_{1}$ and $\:{w}_{2}$ such that there are four actions.

Based on the action, the agent receives a reward that measures the quality of this action. is calculated based on the improvement in the best fitness as follows

17 $$\:{R}_{t}=\:\frac{f\left({x}_{b}^{t-1}\right)-f\left({x}_{b}^{t}\right)}{f\left({x}_{b}^{t-1}\right)}$$

Based on the current state, action taken, and the reward received, the Q-value is updated to reflect the reward for the current action as shown in Eq. 18

18 $$\:Q\left({S}_{t},\:{A}_{t}\right)\leftarrow\:\:Q\left({S}_{t},\:{A}_{t}\right)+\:\alpha\:\:({R}_{t}+\:\gamma\:\:{max}_{A}Q\left({S}_{t+1},{A}_{t}^{{\prime\:}}\right)+Q\left({S}_{t},\:{A}_{t}\right)$$

where $\:\alpha\:$ is the learning rate, γ is the discount factor, and $\:{A}_{t}^{{\prime\:}}$ is the action assigned to the next state $\:{S}_{t+1}$.

Memory-based can efficiently improve the selection of the three points in GOI by leveraging historical information and learning from the previous pattern. The QIQ maintains a memory of the best minimizer ($\:{x}^{*}$) found over iterations, therefore, it can bias the selection of the three points toward areas of historical association with good performance.

Therefore, in the exploration strategy, the calculation of the new minimizer $\:{x}^{*}\left(t\right)$ in Eq. 19 is performed using GOI by randomly selecting two minimizers $\:{x}_{r1}^{*}$ and $\:{x}_{r2}^{*}$ from the historical information and therefore $\:{x}^{*}\left(t\right)$ is expressed as follows.

 

19 $$\:{x}^{\text{*}}\left(t\right)=GOI\:(x\left(t\right),\:{x}_{r1}^{\text{*}}\left(t\right), \:{x}_{r2}^{\text{*}}\left(t\right))$$

Similarly, in the exploitation strategy, the $\:{x}^{\text{*}}\left(t\right)$ is calculated as follows.

20 $$\:{x}^{\text{*}}\left(t\right)=GOI\:({x}_{best}\left(t\right),\:{x}_{r1}^{\text{*}}\left(t\right),{x}_{r2}^{\text{*}}(t))$$

After each iteration, the memory content is refined by replacing the worst stored minimizer $\:{x}^{*}$ with the newly found minimizer $\:{x}^{*}\left(t\right)$ if the new one shows superior fitness.

**Fig. 2:**
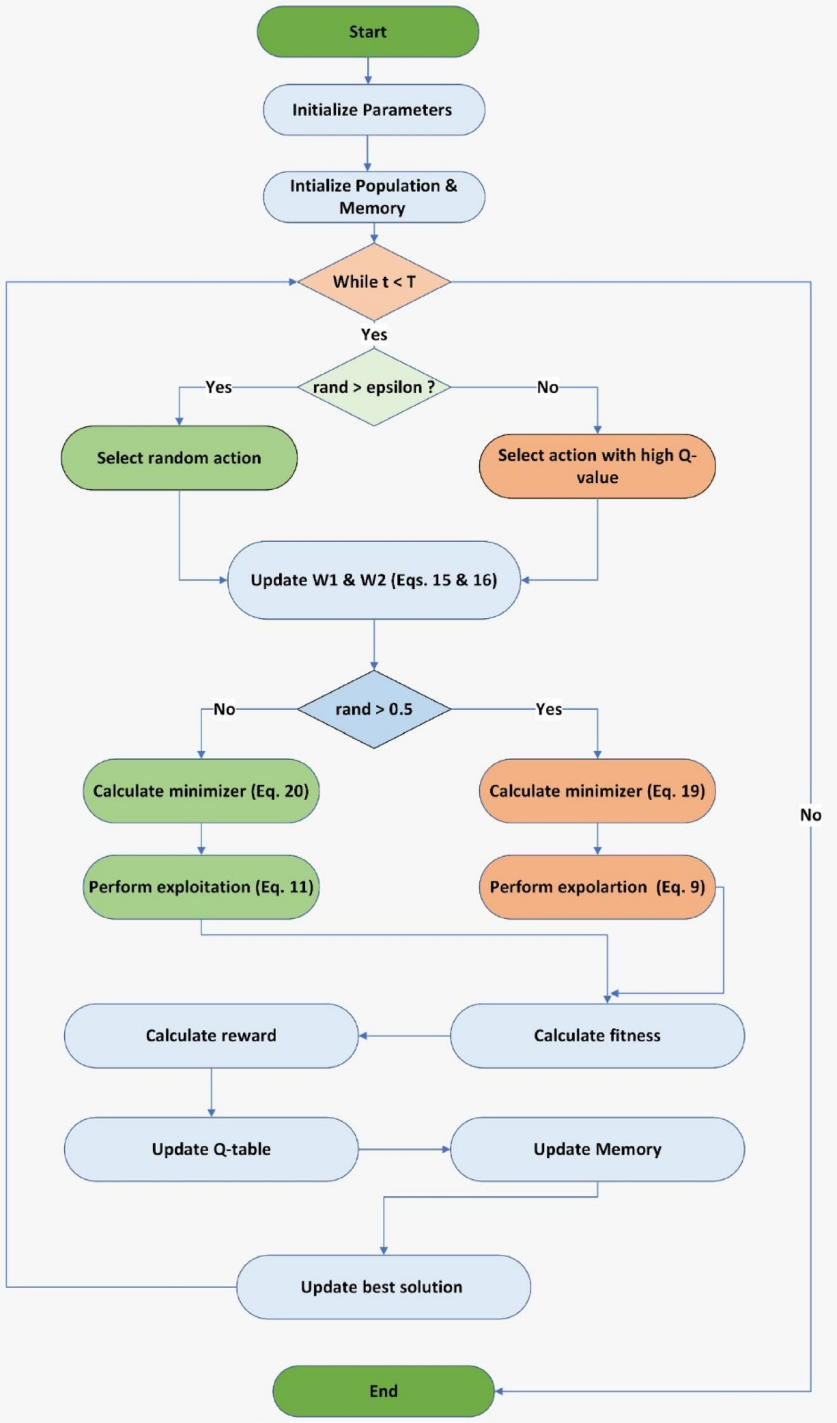
Flowchart of the proposed method MRQIO.

Based on the above, Algorithm (1) shows the pseudocode for the Proposed MRQIQ algorithm and Fig. 2 shows the Flowchart of the proposed method.

The time complexity of Algorithm (1) is determined by the number of populations N, problem dimension D, and number of iterations T. The lines 1–4 initial population and have a time complexity $\:O(N.D)$. The time complexity for lines 5–29 is $\:O\:\left(T.\:N.D\right)$. Therefore, the total time complexity of the algorithm is $\:O\:\left(T.\:N.D\right)$.

In summary A key feature of the MRQIO algorithm is its self-adaptive capability to balance exploration and exploitation. Unlike traditional methods that rely on static parameters, MRQIO dynamically optimizes this balance through its Reinforcement Learning component. The RL agent continuously observes the search state, defined by population diversity and fitness improvement, and autonomously adjusts the exploration and exploitation weights ($\:{w}_{1}$ and $\:{w}_{2}$). This real-time adaptation allows the algorithm to aggressively explore new regions when progress stalls and to intensify local search around promising solutions, thereby ensuring robust convergence without the need for manual parameter tuning.

**Figure:**
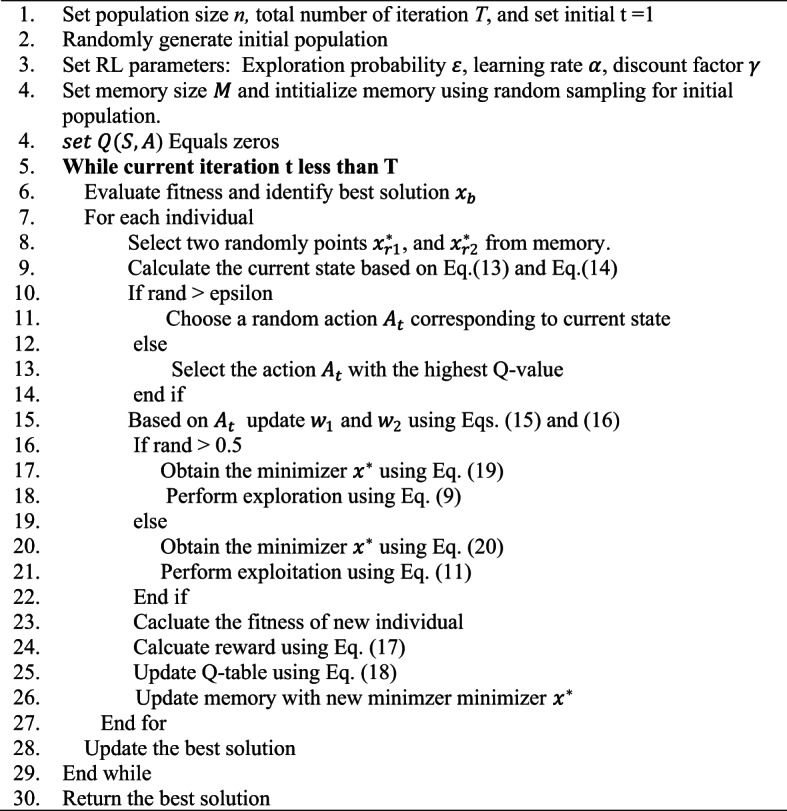
**Algorithm 1: MRQIO.**

## Evaluation metrics and datasets

This section presents the results obtained using the proposed MRQIO approach for estimating the parameters of solar cells. The evaluation criteria used to assess the performance of the optimization technique are outlined as follows:

- **Statistical mean** is calculated according to Eq. 21.

21 $$\:\mathbf{M}\mathbf{e}\mathbf{a}\mathbf{n}=\:\frac{1}{\mathbf{M}}\sum\:_{\mathbf{i}=1}^{\mathbf{M}}{\mathbf{S}}_{\mathbf{i}}$$

where *S*_*i*_ is the obtained solution of the run time *i*.

- **Standard deviation (Std)** is calculated as shown in Eq. 22:

22 $$\:\varvec{S}\varvec{t}\varvec{d}=\:\sqrt{\frac{1}{\varvec{M}-1}\sum\:_{\varvec{i}=1}^{\varvec{M}}{({\varvec{S}}_{\varvec{i}}-\varvec{M}\varvec{e}\varvec{a}\varvec{n})}^{2}}$$

- **Root mean square error (RMSE)** is computed using the following formula:

23 $$\:\varvec{R}\varvec{M}\varvec{S}\varvec{E}=\:\sqrt{\frac{\sum\:_{\varvec{i}=1}^{\varvec{N}}{({\varvec{I}}_{\varvec{m}}-\:{\varvec{I}}_{\varvec{c}})}^{2}}{\varvec{N}}}$$

The RMSE serves as an objective function to assess whether the model, with the estimated parameters, can accurately predict the output of the solar cell.

- **Absolute error (E**_**abs**_**)**: It is the absolute difference between the measured values (*I*_*m*_) and estimated values (*I*_*c*_) as in Eq. (24).

24 $$\:{E}_{abs}=\:\left|{I}_{m}-\:{I}_{c}\right|$$

- **Individual absolute error (IAE)** of the current and power are given in Eq. (25) and Eq. (26).

25 $$\:{IAE}_{Current}=\:\sum\:_{i=1}^{N}\left|{\varvec{I}}_{\varvec{m}}-\:{\varvec{I}}_{\varvec{c}}\right|$$

26 $$\:{IAE}_{Power}=\:\sum\:_{i=1}^{N}\left|{\varvec{P}}_{\varvec{m}}-\:{\varvec{P}}_{\varvec{c}}\right|$$

- **Relative Error (RE)** which is shown in Eq. (27).

27 $$\:\frac{\left({\varvec{I}}_{\varvec{m}}-\:{\varvec{I}}_{\varvec{c}}\right)}{{\varvec{I}}_{\varvec{m}}}$$

Five PV modules were used in the experimental tests are R.T.C France (SDM and DDM), Photowatt-PWP201, STM6-40/36, and STP6-120/36 cell/modules^50,51^. The configuration of the lower bound (LB) and upper bound (UP) of the PV modules as shown in Table 1.

**Table 1:** Parameter range of PV modules^52^.

Parameters	Single Diode		Double Diode		STM6-40/36		STP6-120/36		Photowatt-PWP201	
	LB	UB	LB	UB	LB	UB	LB	UB	LB	UB
$\:{I}_{ph}\left(A\right)$	0	1	0	1	0	2	0	8	0	2
$\:{I}_{0}\left(\mu\:A\right)/{I}_{01}\left(\mu\:A\right)$, $\:{I}_{02}\left(\mu\:A\right)$	0	1	0	1	0	50	0	50	0	50
$\:{I}_{sh}\left(\varOmega\:\right)$	0	0.5	0	0.5	0	0.36	0	0.36	0	2
$\:{R}_{s}\left(\varOmega\:\right)$	0	100	0	100	0	1000	0	1500	0	2000
$\:N/{N}_{1},\:{N}_{1}$	1	2	1	2	1	60	1	50	0	50

## Experimental results and discussion

The simulations were conducted using MATLAB 2014b on a Dell Latitude E6540 laptop with 16 GB of RAM. The proposed MRQIQ algorithm was evaluated using 13 benchmark functions from the CSE 2022 benchmark suite. Besides, Four types of solar cells were considered for parameter optimization using the MRQIQ algorithm: RTC SDM, RTC DDM, STP6-120/36, STM6-40/3, and PWP201. The detailed parameters of these solar modules are summarized in Table 1. For each solar cell module, the parameters were initialized with realistic bounds based on manufacturer specification and shown in Table 1. The MRQIQ algorithm iteratively adjusts these parameters with monitoring convergence through changes in the RMSE value ensuring efficient and reliable exploration and exploitation of the parameter space.

The performance of MRQIO was compared to the performance of relevant work in the literature review such as QPSOL^52^, HOA^53^, TLBO^54^, GA^55^, DE^56^, PSO^57^, ABC^58^, GWO^59^, SCA^60^, BBO^61^, ACO^62^,, SA^28^, HS^30^, COVIDOA^63^, QIO^64^, RCSA^65^, and KMA2D^66^.

For MRQIO, the memory $\:M=\:0.1\text{*}N\:$(20% of the population size), $\:\epsilon\:$ epsilon start with 0.8 to enforce exploration and reduce over iterations, learning rate $\:\alpha\:$ is set to 0.1 to provide stability and smoothness convergence, and the discount factor $\:\gamma\:$ is set to 0.9 to balance immediate and future reward.

### Performance evolution on CSE benchmark

The performance of the proposed MRQIQ algorithm was assessed using 13 benchmark functions from the CSE 2022 benchmark suite. These functions are categorized into unimodal functions (F1–F7) and multimodal functions (F8–F13). Detailed descriptions of the benchmark set can be found in^53^. Table 2 show that the proposed MRQIQ demonstrate superior performance as it achieves near-zero mean values, indicating that it successfully reaches global minimum and stable performance across most of benchmark functions. In contrast, ACO, SCA, and DE exhibit much higher mean values as well as standard deviation values which indicate slower convergence and instability.

Based on the results presented in Table 2, which evaluates algorithm performance on unimodal benchmark functions (F1-F7), the proposed MRQIO algorithm demonstrates exceptionally strong and stable performance. For functions F1 through F4, MRQIO achieves mean values that are effectively zero, matching or coming extremely close to the theoretical global optimum. This performance is on par with the top-performing algorithms in the comparison, namely QIO and KMA2D, indicating that MRQIO has inherited and maintained the powerful local search capabilities of the original QIO algorithm. The ability to consistently hit the true minimum on these unimodal functions, which are designed to test an algorithm’s exploitation and convergence speed, is a clear sign of an efficient and precise optimization method.

For F6, MRQIO continues to perform excellently, maintaining a near-zero mean. For F5 and F7, while MRQIO’s performance is still highly competitive, it is slightly surpassed by the standard QIO algorithm. QIO achieves a lower mean value on both F5 (3.05E-06 vs. 3.203099) and F7 (0.000836 vs. 0.001127). This suggests that the enhancements introduced in MRQIO, the reinforcement learning and memory mechanisms, while beneficial for overall balance and preventing local optima, can introduce a minor overhead or a different search dynamic that very slightly reduces peak exploitation performance on a couple of specific, highly complex unimodal problems compared to the pure QIO approach.

Despite this minor trade-off on two functions, the overall performance of MRQIO on the unimodal benchmark suite is outstanding. The algorithm consistently ranks among the very best, significantly outperforming a wide range of established metaheuristics like GA, PSO, DE, and GWO by many orders of magnitude. The low standard deviation values for MRQIO, particularly on F1-F4, further confirm the stability and reliability of its results across multiple independent runs. This robust performance on unimodal functions validates that MRQIO is a highly effective and precise optimizer, laying a strong foundation for its application to more complex, real-world problems like PV parameter estimation, which requires both accuracy and reliability.

Table 3 presents the average and standard deviation values of MRQIQ’s performance compared with other competing algorithms. The comparative analysis reveals that MRQIQ consistently outperforms the classical optimization methods across all tested functions. Its low mean and small standard deviation values indicate efficient exploration of the search space and stable convergence behavior. In contrast, algorithms such as GA, DE, and PSO exhibit higher variability in both their mean and standard deviation results, reflecting less consistent performance.

Table 3, which details algorithm performance on multimodal benchmark functions (F8-F13), reveals a more complex and telling story about the MRQIO algorithm’s capabilities compared to the unimodal results in Table 2. Unlike unimodal functions, multimodal functions contain numerous local optima, specifically testing an algorithm’s ability to explore the search space and avoid premature convergence. In this challenging context, MRQIO demonstrates a powerful capacity for exploration, but not without observable trade-offs. It achieves perfect results on F11, matching several other algorithms, and delivers an exceptionally low mean on F10 (1.53E-12), showcasing its ability to navigate complex search spaces and find high-quality solutions where many classical algorithms like GA, PSO, and ABC fail significantly.

However, the results also highlight the specific performance characteristics imparted by its hybrid design. On functions like F8 and F9, where the original QIO and HOA algorithms achieve the theoretical optimum, MRQIO converges to good but not perfect solutions. This is a direct consequence of the reinforcement of learning and memory-based mechanisms; these components promote diversity and prevent stagnation in deceptive landscapes, but they can also prevent the algorithm from performing the ultra-fine, final-step exploitation that leads to a perfect score on some of these specific benchmarks. This is not necessarily a weakness but rather an indication that MRQIO’s strength lies in robust global search rather than pure, peak exploitation on every single function type.

Ultimately, the performance of MRQIO on this multimodal suite must be interpreted in the context of its intended application. For real-world, complex problems like PV parameter estimation, which is inherently multimodal and noisy, the ability to consistently find a very-near-optimal solution is more valuable than occasionally hitting a perfect score on a synthetic benchmark. While algorithms like QIO and HOA show superior results on a few specific functions, MRQIO’s performance is far more robust and superior across the broader range when compared to most competitors like DE, PSO, GWO, and SCA. Its strong and balanced performance in Table 3 validates the core contribution of the paper: that the MRQIO enhancements successfully equip the algorithm with a robust exploration mechanism, making it highly suitable for the complex, multi-modal optimization problems it was designed to solve.

**Table 2:** Performance evaluation of mrqio on unimodal benchmark functions.

Functions		F1	F2	F3	F4	F5	F6	F7
HOA	mean	1.57E-23	1.0254E-11	2.523E-17	8.424E-12	52.79214	22.86235	0.000893
	std	2.68E-23	1.048E-11	7.835E-17	4.761E-12	27.47709	0.613624	0.000569
TLBO	mean	3.23E-79	1.8452E-40	6.879E-08	6.488E-33	97.18999	4.7486367	0.001633
	std	3.49E-79	1.5683E-40	2.169E-07	5.767E-33	0.633953	0.9273714	0.000731
GA	mean	264339.2	3.9691E + 43	1332471.2	94.334597	1.13E + 09	259,878	633.6712
	std	13703.05	2.1162E + 44	473386.44	1.3576305	1.07E + 08	12341.61	53.4546
DE	mean	8953.785	38.47216	151136.72	96.767483	10,905,872	7774.941	7.949219
	std	3436.716	9.59201507	32741.859	1.033392	6,712,352	2065.5266	3.604812
PSO	mean	60097.12	298.083467	171722.1	54.959493	64,979,323	59712.153	73.82664
	std	11632.4	30.3207306	50464.711	3.9384859	34,017,716	12222.256	23.9214
ABC	mean	107122.4	250.742467	416846.27	92.199877	3.27E + 08	105222.32	286.6879
	std	15920.14	410.372787	56687.452	1.0255238	89,040,848	16002.293	60.486
GWO	mean	437.0467	0.8877231	184808.86	91.198157	359343.7	465.6881	1.066187
	std	263.5857	0.57114709	43829.576	5.407753	263,667	427.01016	0.381807
SCA	mean	15026.55	10.1374994	268409.5	90.60493	1.25E + 08	13609.811	61.87501
	std	10802.36	7.77603087	59281.2	2.3033783	66,120,730	7418.9011	27.64327
BBO	mean	282.1683	13.0291967	57291.323	31.32247	7506.177	279.82187	0.21825
	std	28.08259	1.17852186	8746.1469	2.4713499	1318.009	28.113792	0.042674
ACO	mean	172228.2	5.7931E + 33	652788.97	96.854443	1.22E + 09	175264.43	493.9246
	std	12252.2	3.0654E + 34	86333.613	1.1540279	88,454,954	13480.106	71.10298
RCSA	mean	0.000149	3.0798906	0.0225141	0.0050566	138.495	0.0001523	0.088183
	std	1.2E-05	11.6908599	0.0024261	0.000458	77.11081	1.292E-05	0.023819
HS	mean	28849.62	81.9295967	408066.37	65.334303	46,271,160	28763.893	23.93719
	std	3436.43	5.4527525	63101.366	1.6374134	7,601,010	2700.1316	3.304454
COVIDOA	mean	36710.38	136.158133	407,641	89.285547	51,218,157	35475.343	27.20487
	std	5090.537	11.0313099	66868.426	9.7336226	11,486,758	4270.5374	7.210926
KMA2D	mean	0	0	0	0	98.82898	18.10667	0.000854
	std	0	0	0	0	0.099221	3.3119338	0.000852
QIO	mean	0	2.927E-297	0	8.65E-293	3.05E-06	1.099E-07	0.000836
	std	0	0	0	0	4.39E-06	5.093E-08	0.000722
MRQIQ	mean	0	0	4.2E-298	5.43E-273	3.203099	1.017E-07	0.001127
	std	0	0	0	0	17.54408	3.558E-08	0.000795

**Table 3:** Performance evaluation of MRQIO on multimodal benchmark functions.

Function		F8	F9	F10	F11	F12	F13
HOA	mean	−41898.3	0	8.88E-16	0	6.99E-09	4.21E-08
	std	0	0	0	0	2.58E-09	1.35E-08
TLBO	mean	−16,613	6.689567	7.76E-15	0	0.058123	6.246401
	std	3478.211	36.64027	9.01E-16	0	0.026351	0.90177
GA	mean	−6907.74	1484.045	20.69692	2323.981	24.18157	103.7302
	std	808.6695	42.68097	0.068865	120.5435	1.943128	6.106548
DE	mean	−14426.5	534.6371	11.48715	80.15497	0.564611	4.869307
	std	3306.243	165.5609	1.490385	25.05753	0.219572	1.441781
PSO	mean	−16567.4	708.317	18.54249	492.6265	5.177229	40.48198
	std	1909.762	74.9949	0.490661	129.9769	0.854019	6.176659
ABC	mean	−16490.9	983.5111	19.79322	1022.903	10.85817	46.79276
	std	867.8853	66.13227	0.236479	192.8635	1.438922	7.073009
GWO	mean	−9627.7	828.2916	20.8953	6.307807	3.807776	5.236407
	std	1073.028	86.25987	0.09827	3.47694	1.585343	0.77821
SCA	mean	−6938.86	289.3135	18.26415	110.1992	3.312723	13.58429
	std	541.9982	111.4916	4.196379	77.61881	1.683859	1.839454
BBO	mean	−21671.5	391.5791	3.71943	3.58902	0.82007	0.117623
	std	1220.565	46.41371	0.138159	0.26695	0.430857	0.015696
ACO	mean	−8285.2	1446.495	20.71817	1572.491	23.26834	56.71944
	std	617.2058	40.24589	0.029607	120.07	1.663696	3.801377
RCSA	mean	−31432.1	255.107	0.001603	0.001356	0.004147	0.00293
	std	928.0863	32.31266	6.71E-05	0.003299	0.010753	0.004942
HS	mean	−33710.7	357.6258	14.92513	259.8599	2.14402	11.21998
	std	663.8899	23.62106	0.327604	29.51746	0.340641	1.102371
COVIDOA	mean	−12078.1	925.9183	15.96087	327.5766	4.702846	14.64272
	std	1160.923	44.84391	0.453723	45.47594	0.641235	1.873446
KMA2D	mean	−17482.1	0	8.88E-16	0	0.261882	3.450161
	std	5820.659	0	0	0	0.106586	1.573063
QIO	mean	−41898.3	0	8.88E-16	0	7.36E-09	3.86E-08
	std	0	0	0	0	2.41E-09	1.09E-08
MRQIQ	mean	−7914.03	128.0862	1.53E-12	0	1.013451	0.654452
	std	980.6392	239.306	1.58E-12	0	0.100519	0.200127

### *Experimental* series *1: RTC France solar cell (SDM)*

Table 4 presents a comparative analysis of calculated and measured values for the RTC France (SDM) photovoltaic module, highlighting the performance of parameter estimation methods. The close values between the measured current and calculated current, with minimal IAE for current values, indicate the reliability of MRQIO. Similarly, the calculated power and measured power align closely, with a consistently low IAE for power values, underscoring the accuracy of the model in predicting the module’s performance. Overall, the low error metrics confirm the robustness of the parameter estimation using MRQIO, validating its application for modeling PV modules under varying conditions.

Table 5 provides a comparative analysis of the performance of various algorithms applied to the parameter estimation of RTC France (SDM). The MRQIO algorithm demonstrated the lowest RMSE (both Std and Mean), indicating superior accuracy and consistency in parameter estimation. In contrast, other comparative algorithms showed higher RMSE values, suggesting relatively less precision in estimating parameters.

**Fig. 3:**
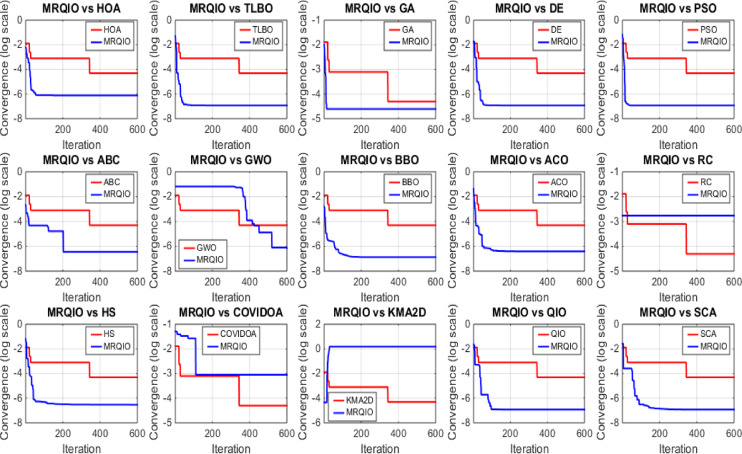
Convergence behavior of MRQIO compared with other optimization algorithms for RTC France (SDM).

Figure 3 provides a critical visual representation of the convergence behavior of the proposed MRQIO algorithm against a suite of state-of-the-art optimization methods over 600 iterations for the RTC France SDM. The log-scale plot of the objective function (RMSE) reveals not just the superiority of MRQIO, but the underlying mechanics of its enhanced design. MRQIO demonstrates a characteristically steep descent in the initial phase (approximately the first 50–100 iterations), rapidly navigating the search space to locate a highly promising region. This aggressive early convergence is a direct benefit of its reinforcement learning component, which dynamically allocates resources to exploration when the population is diverse and fitness improvements are high, allowing it to quickly discard non-optimal areas of the search space that trap slower-converging algorithms.

The performance gap between MRQIO and other algorithms is both significant and informative. Classical algorithms like GA, PSO, and DE show a much slower, more gradual decline, often stagnating at a higher RMSE. This indicates their susceptibility to premature convergence or an inefficient balance between exploration and exploitation. Notably, algorithms like ABC and GWO, while showing a steeper initial drop than GA or PSO, still plateau well above MRQIO’s final value. This suggests that while they possess strong exploration capabilities, they lack the sophisticated local refinement mechanism that MRQIO inherits from the QIO core. QIO’s quadratic interpolation provides a powerful, derivative-free method for local exploitation, and when guided by the RL and memory-based mechanisms, this exploitation is directed far more effectively.

Furthermore, the stability of the MRQIO curve after convergence is as important as its speed. The flat, low-value plateau it maintains for most of the iterations indicates a robust and stable solution, with no late-iteration divergence. This stability can be attributed to the memory-based mechanism, which preserves high-quality solutions (“minimizers”) from previous iterations. This archive of historical information prevents the algorithm from regressing and helps maintain a diverse population that is biased towards productive regions of the search space. In conclusion, Fig. 3 does not merely show that MRQIO is faster; it visually validates the synergistic success of its hybrid architecture, the RL-driven exploration quickly finds the promising basin, and the memory-guided QIO exploitation thoroughly and stably refines the solution to a degree unattainable by the competing algorithms.

The parameter space exploration and optimization process in MRQIO was specifically engineered to ensure efficient convergence through a tripartite strategy that dynamically balances global search and local refinement. First, the Reinforcement Learning component acts as an adaptive controller. By continuously monitoring the state of the optimization—defined by population diversity and fitness improvement, the RL agent intelligently adjusts the exploration and exploitation weights ($\:{w}_{1}$ and $\:{w}_{2}$). This ensures that the algorithm aggressively explores undiscovered regions when progress is stagnant and switches to intensive local search when a promising area is identified, thereby preventing premature convergence.

Second, the memory-based mechanism enhances exploration quality by biasing the selection of points for the Quadratic Interpolation (GQI) towards historically successful ‘minimizers’ stored in memory. This guides the search more efficiently than random selection, learning from past patterns to focus computational effort on fruitful regions of parameter space. Finally, the core QIO exploitation strategy provides a robust and low-noise local search, using quadratic interpolation to rapidly converge to a precise minimum within a identified promising region. The synergy between these three components, RL-driven adaptation, memory-guided exploration, and interpolation-based exploitation, creates a feedback loop that systematically navigates the complex, multimodal parameter space of PV models, which is the fundamental reason for MRQIO’s superior convergence speed and accuracy.

**Table 4:** Comparison of calculated and measured values for RTC France (SDM) module.

S.no	$\:{I}_{m}$	$\:{I}_{e}$	IAE(Current)	V	$\:{P}_{m}$	$\:{P}_{e}$	IAE (power)
1	0.764	0.76406	0.000622	−0.2057	−0.1571548	−0.15717	1.28098E-05
2	0.762	0.76265	0.000654	−0.1291	−0.0983742	−0.09846	8.44497E-05
3	0.7605	0.76136	0.000861	−0.0588	−0.0447174	−0.04477	5.06557E-05
4	0.7605	0.76017	0.000325	0.0057	0.00433485	0.00433	1.85786E-06
5	0.76	0.75909	0.000912	0.0646	0.049096	0.04904	5.89212E-05
6	0.759	0.75809	0.000913	0.1185	0.0899415	0.08983	0.000108254
7	0.757	0.75715	0.000145	0.1678	0.1270246	0.12705	2.44714E-05
8	0.757	0.7562	0.000796	0.2132	0.1613924	0.16122	0.000169723
9	0.7555	0.75516	0.000344	0.2545	0.19227475	0.19219	8.7779E-05
10	0.754	0.75373	0.000266	0.2924	0.2204696	0.22039	7.79074E-05
11	0.7505	0.75146	0.000955	0.3269	0.24533845	0.24565	0.000312419
12	0.7465	0.7474	0.000904	0.3585	0.26762025	0.26794	0.000324365
13	0.7385	0.74014	0.001643	0.3873	0.28602105	0.28666	0.000636688
14	0.728	0.72737	0.000625	0.4137	0.3011736	0.30091	0.000258616
15	0.7065	0.70693	0.000427	0.4373	0.30895245	0.30914	0.000186795
16	0.6755	0.6752	0.000299	0.459	0.3100545	0.30992	0.000137478
17	0.632	0.63066	0.00134	0.4784	0.3023488	0.30171	0.000641085
18	0.573	0.57183	0.001166	0.496	0.284208	0.28363	0.000578613
19	0.499	0.49954	0.000537	0.5119	0.2554381	0.25571	0.000275087
20	0.413	0.41362	0.000622	0.5265	0.2174445	0.21777	0.000327671
21	0.3165	0.31753	0.001031	0.5398	0.1708467	0.1714	0.000556547
22	0.212	0.21222	0.000215	0.5521	0.1170452	0.11716	0.000119073
23	0.1035	0.10233	0.001170	0.5633	0.05830155	0.05764	0.000659211
24	−0.01	−0.0086	0.001337	0.5736	−0.005736	−0.00497	0.000767192
25	−0.123	−0.1255	0.002504	0.5833	−0.0717459	−0.07321	0.001461069
26	−0.21	−0.2085	0.001447	0.59	−0.1239	−0.12305	0.000853875

**Table 5:** A comparative analysis of different algorithms applied to the single diode model (SDM).

Algorithms	RMSE			$\:{I}_{ph}$	$\:{I}_{sd}$	$\:{R}_{s}$	$\:{R}_{sh}$	$\:n$
	Std	Mean	Minimum					
HOA	0.031472	0.02776	0.002211	0.761750847	0.814104059	0.032384175	69.01928784	1.580699309
TLBO	0.001049	0.00009	0.000986	0.760784623	0.325758883	0.036339076	53.71161908	1.482039798
GA	0.101096	0.06564	0.009971	0.753835719	0.617494607	0.027993571	34.02415376	1.551960241
DE	0.001413	0.00029	0.000988	0.760760614	0.33395053	0.036244776	54.63855555	1.484541233
PSO	0.047488	0.08854	0.000987	0.760768625	0.32818079	0.036311141	54.16091442	1.482780544
ABC	0.004832	0.00168	0.001580	0.759117605	0.331767452	0.035790253	63.44589148	1.483897722
GWO	0.016761	0.01534	0.002384	0.758146416	0.522775456	0.034378175	47.34119057	1.531286643
SCA	0.050251	0.03444	0.013496	0.773684172	0.92845157	0.023605372	11.8749308	1.601811736
BBO	0.006027	0.00757	0.001032	0.760700787	0.378866744	0.035739496	58.55892991	1.4974107
ACO	0.0023396	0.00016	0.001650	0.760479308	0.640449793	0.033530572	83.56452548	1.553433525
RCSA	0.147397	0.02526	0.063149	0.805492287	0.085712027	0.038265368	97.82373688	1.362328166
HS	0.002115	0.00033	0.001452	0.760832204	0.554656656	0.034084136	67.3437749	1.537762771
COVIDOA	0.123051	0.04747	0.047383	0.754908916	1	0	12.01685382	1.608602384
KMA2D	0.219601	0.17967	0.013029	0.752321549	0.000724143	0.054879547	59.01314032	1.045426761
QIO	0.000992	0.00092	0.000986	0.760769446	0.322759381	0.03637891	53.73401553	1.481101818
QPSOL	2.2054E-19	0.00099	0.007896	0.76077553	3.23021E-07	0.036377092	5.3718537E + 01	1.4811836
MRQIO	0.000988	0.000003	0.000986	0.760786856	0.332298463	0.036265397	54.34759911	1.484040498

**Fig. 4:**
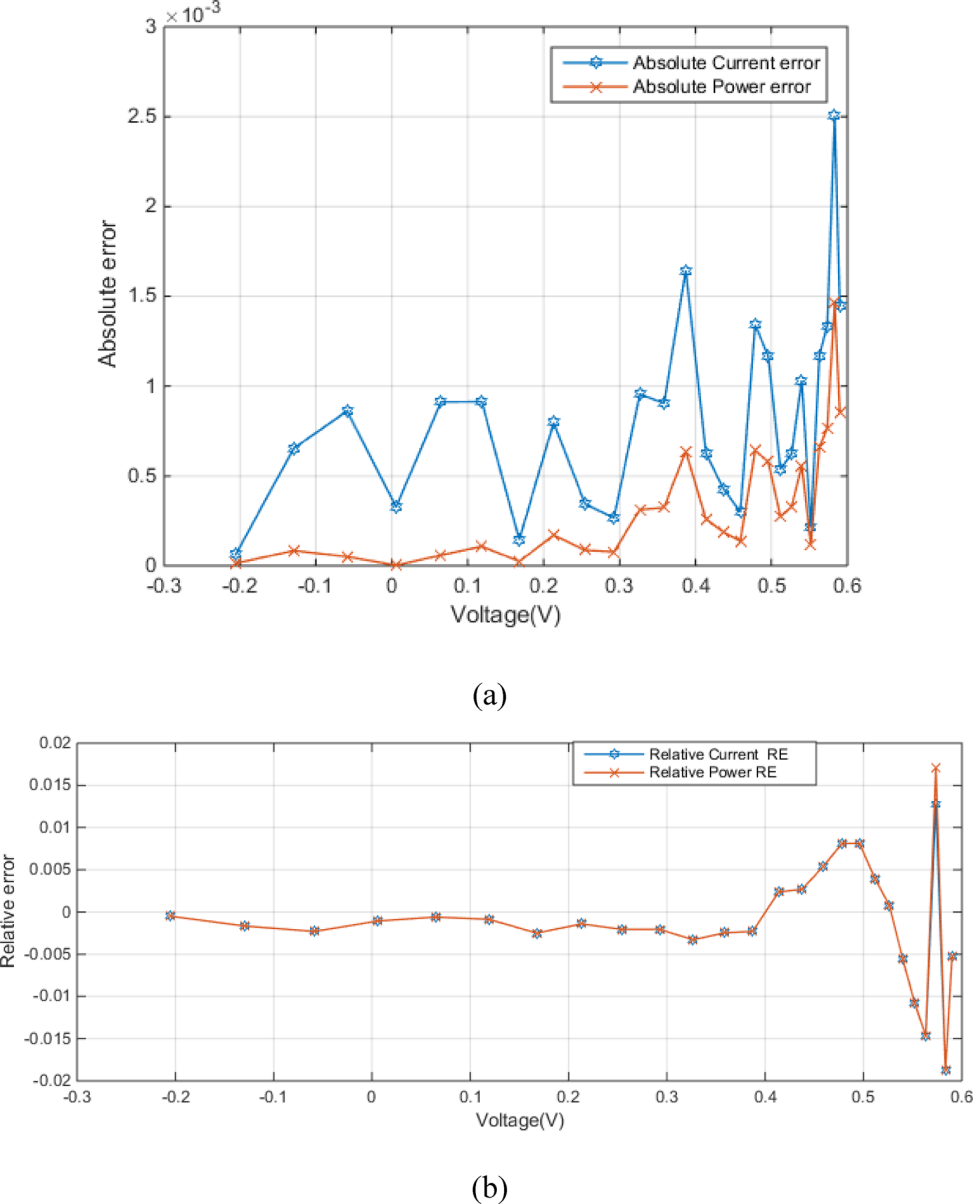
Error-index values for experimental and simulated current data in the SDM (**a**) (IAE) (**b**) (RE).

Figure 4 (a) shows the absolute current and power errors as a function of voltage for RTC France (SDM). The plot highlights the voltage-dependent accuracy of the SDM, especially concerning current prediction at higher voltages. Figure 4 (b) shows the relative current error (RE) and relative power error (RE) as a function of voltage. Both errors are generally small within the voltage range of approximately − 0.2 V to 0.4 V, fluctuating around zero. This indicates a good match between the modeled and measured values in this region. The relative power error exhibits larger magnitudes in this region. The close tracking of both error types suggests a strong correlation between them, implying that inaccuracies in current prediction directly influence the power calculation.

**Fig. 5:**
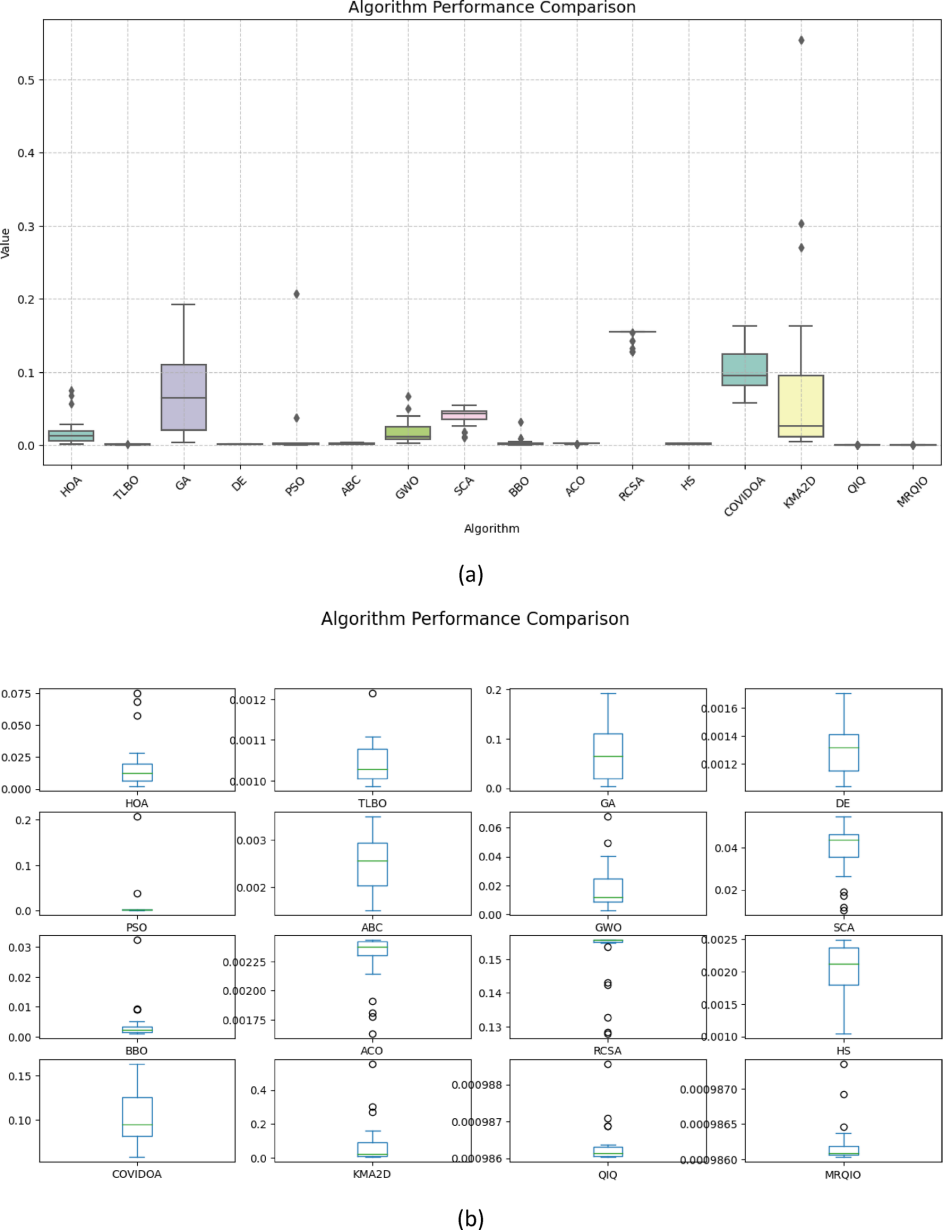
Algorithm Performance Comparison Using RMSE.

Figure 5 provides a compelling visual demonstration of MRQIO’s superior convergence characteristics for the single diode model (SDM). The plot clearly shows that MRQIO achieves a significantly faster initial descent in RMSE compared to all other algorithms, indicating its exceptional efficiency in navigating the complex, high-dimensional parameter space to rapidly locating promising regions.

Furthermore, MRQIO converges to a lower final RMSE value than its competitors, including the standard QIO, underscoring how the integration of reinforcement learning and memory mechanisms enhances both the speed and precision of the search process. This consistent ability to avoid local optima and refine solutions to a high degree of accuracy validates the algorithm’s robust balance between exploration and exploitation, making it particularly effective for the challenging task of multi-diode model parameter estimation.

### Experimental series 2: RTC france solar cell (DDM)

Table 6 presents a comparative analysis of calculated and measured values for the RTC France (DDM) photovoltaic module, highlighting the performance of parameter estimation methods. As shown in Table 6, MRQIO algorithm demonstrates a strong ability to accurately model the RTC France (DDM) module, with minimal differences between the calculated and measured current (I) and power (P) values. The low values of the Integral Absolute Error (IAE) for both current and power across all data points indicate a consistent level of accuracy in the algorithm’s performance. Besides, the algorithm maintains its accuracy across a wide range of voltage (V) values, demonstrating its robustness and applicability under varying operating conditions. Overall, the results presented in Table 6 strongly support the effectiveness of the MRQIO optimization algorithm for accurately modeling the RTC France (DDM) module. Its high accuracy, consistency, and robustness make it a promising tool for the optimization of photovoltaic systems.

**Table 6:** Comparison of calculated and measured values for RTC France (DDM) module.

S.no	$\:{I}_{m}$	$\:{I}_{e}$	IAE(Current)	V	$\:{P}_{m}$	$\:{P}_{e}$	IAE (power)
1	0.7639	0.7629	0.001049	−0.2057	−0.15714	−0.15693	0.000216
2	0.7626	0.7621	0.000508	−0.1291	−0.09845	−0.09838	0.000066
3	0.7613	0.7613	0.000013	−0.0588	−0.04477	−0.04477	0.000001
4	0.7602	0.7606	0.000442	0.0057	0.00433	0.00434	0.000003
5	0.7591	0.76	0.000857	0.0646	0.04904	0.0491	0.000055
6	0.7582	0.7594	0.001234	0.1185	0.08984	0.08999	0.000146
7	0.7572	0.7588	0.001572	0.1678	0.12707	0.12733	0.000264
8	0.7563	0.7582	0.001858	0.2132	0.16124	0.16164	0.000396
9	0.7552	0.7573	0.002059	0.2545	0.1922	0.19273	0.000524
10	0.7537	0.7558	0.002118	0.2924	0.22039	0.22101	0.000619
11	0.7513	0.7533	0.001944	0.3269	0.24561	0.24625	0.000636
12	0.7472	0.7486	0.001431	0.3585	0.26786	0.26837	0.000513
13	0.7398	0.7403	0.000502	0.3873	0.28654	0.28673	0.000194
14	0.7271	0.7263	0.000833	0.4137	0.30081	0.30046	0.000345
15	0.7068	0.7044	0.002336	0.4373	0.30907	0.30805	0.001021
16	0.6753	0.6716	0.003652	0.459	0.30996	0.30829	0.001676
17	0.6311	0.6268	0.004274	0.4784	0.30191	0.29987	0.002045
18	0.5724	0.5685	0.003878	0.496	0.28391	0.28198	0.001923
19	0.4998	0.4973	0.002437	0.5119	0.25584	0.25459	0.001247
20	0.4136	0.4134	0.00026	0.5265	0.21778	0.21764	0.000137
21	0.3172	0.3192	0.002026	0.5398	0.1712	0.1723	0.001094
22	0.2119	0.2156	0.003719	0.5521	0.11697	0.11902	0.002053
23	0.1024	0.1065	0.004106	0.5633	0.05769	0.06	0.002313
24	−0.0095	−0.0068	0.002681	0.5736	−0.00544	−0.0039	0.001538
25	−0.1244	−0.1254	0.001018	0.5833	−0.07254	−0.07313	0.000594
26	−0.2089	−0.2142	0.005336	0.59	−0.12323	−0.12638	0.003148

**Table 7:** A comparative analysis of different algorithms applied to the double diode model (DDM).

Algorithms	RMSE			$\:{I}_{ph}$	$\:{I}_{sd1}$	$\:{R}_{s}$	$\:{R}_{sh}$	$\:{n}_{1}$	$\:{I}_{sd2}$	$\:{n}_{2}$
	Std	Mean	Minimum							
HOA	0.0459	0.0287	0.00516	0.7651	0.1843	0.0276	36.3821	1.7075	1.5	1.6664
TLBO	0.0016	0.0008	0.00030	0.7607	0.1954	0.0368	54.3359	1.4388	1	2
GA	0.1113	0.1061	0.00552	0.7632	0.127	0.032	26.5275	1.4225	1.1802	1.8027
DE	0.0026	0.0007	0.00101	0.7606	0.288	0.035	73.1592	1.48	1	1.9336
PSO	0.0666	0.0929	0.00031	0.7609	0.086	0.0385	53.0167	1.3704	1.8797	2
ABC	0.0051	0.0013	0.00279	0.7579	0.1945	0.0331	100	1.4588	1.3556	1.8365
GWO	0.0191	0.0118	0.00470	0.7618	0.5003	0.0275	67.1552	1.5769	1.3352	1.7691
SCA	0.032	0.0091	0.01166	0.7581	0	0.0328	65.9923	2	1.7584	1.6769
BBO	0.0043	0.0021	0.00105	0.7609	0.3048	0.0349	68.041	1.4845	1	1.9561
ACO	0.0032	0.0120	0.00218	0.7609	0.6434	0.0322	100	1.5618	1.0022	2
RC	0.229	0.1843	0.04995	0.7048	0.4531	0.0351	92.1335	1.5438	1.0453	1.8543
HS	0.004	0.0854	0.00254	0.7611	0.4785	0.0314	98.7646	1.5508	1.0345	1.8019
COVIDOA	0.0856	0.0207	0.02925	0.7288	0	0.0162	32.9358	1.8363	1.6631	1.6769
KMA2D	0.1615	0.0821	0.01633	0.7651	0.1988	0.0407	39.0523	1.4845	1.0488	1.7283
QIO	0.052	0.0020	0.00025	0.7607	0.2036	0.0367	58.891	1.4431	1.0414	1.9967
QPSOL	3.4E-06	0.001	0.03215	0.7607	7.47E-07	0.5548	1.999	2.2E-07	3.67E-02	1.4511
MRQIO	0.0019	0.0007	0.00034	0.7609	0.0041	0.0311	93.7746	1.5907	1.005	1.6057

Table 7 presents a comparative analysis of various optimization algorithms applied to RTC France model (DDM) for photovoltaic (PV) parameter extraction. MRQIO has one of the lowest RMSE values (both mean and standard deviation) among all the algorithms. The low RMSE of MRQIO indicates that the parameters it extracts from the DDM result in a very accurate model, closely matching the actual behavior of the PV module. MRQIO’s performance is very close to TLBO and QIO, which are considered top performers in this comparison. These algorithms also have very low RMSE values. The low standard deviation of MRQIO’s RMSE suggests that the algorithm is stable and consistently produces accurate results across different runs or datasets.

**Fig. 6:**
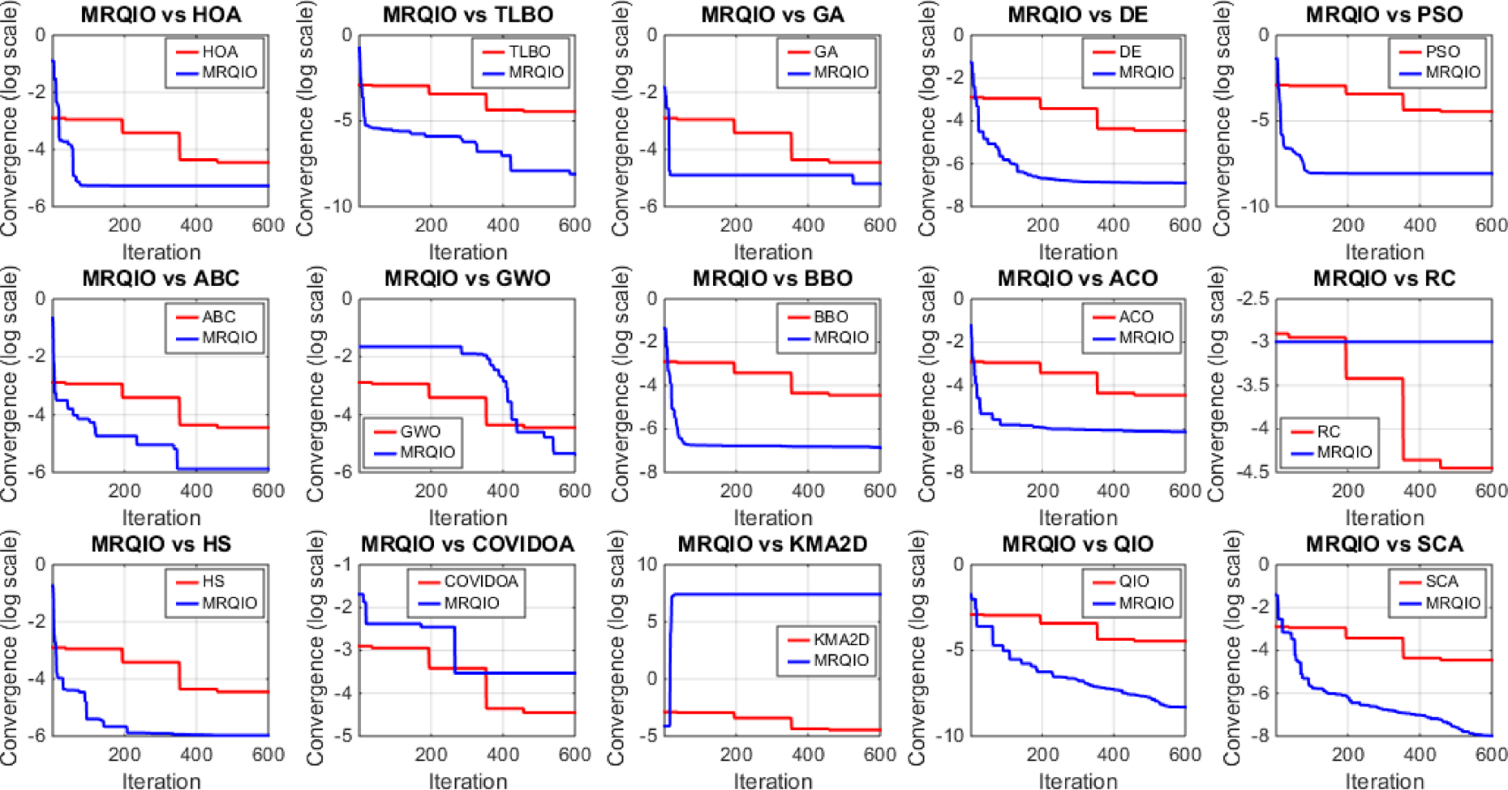
Convergence behavior of MRQIO compared with other optimization algorithms for RTC France (DDM).

Figure 6 offers a critical evaluation of the MRQIO algorithm’s performance on the more complex RTC France double diode model (DDM), which presents a seven-dimensional parameter space. The convergence plot demonstrates MRQIO’s decisive superiority, characterized by a remarkably steep initial decline in RMSE. This rapid descent in the early iterations indicates that the algorithm’s hybrid mechanism, combining reinforcement learning for adaptive decision-making with a memory of past solutions, is exceptionally effective at navigating the intricate search space.

It quickly discards suboptimal regions and identifies a highly promising basin of attraction, a task where algorithms like GA, PSO, and ABC struggle significantly, as evidenced by their slower, more gradual convergence. The latter phase of the convergence curve further solidifies MRQIO’s advantages. Not only does it converge faster, but it also achieves a final RMSE value lower than all other algorithms, including its predecessor, QIO. This indicates that the proposed enhancements do not merely accelerate the search but also refined its quality, enabling more precise local exploitation once a promising region is found.

The performance gap with other well-established algorithms like TLBO and BBO is particularly telling, highlighting MRQIO’s robust balance between global exploration and local refinement. Ultimately, Fig. 6 validates that the memory-based reinforcement learning framework is uniquely capable of handling the increased complexity and multimodality of the DDM parameter extraction problem.

**Fig. 7:**
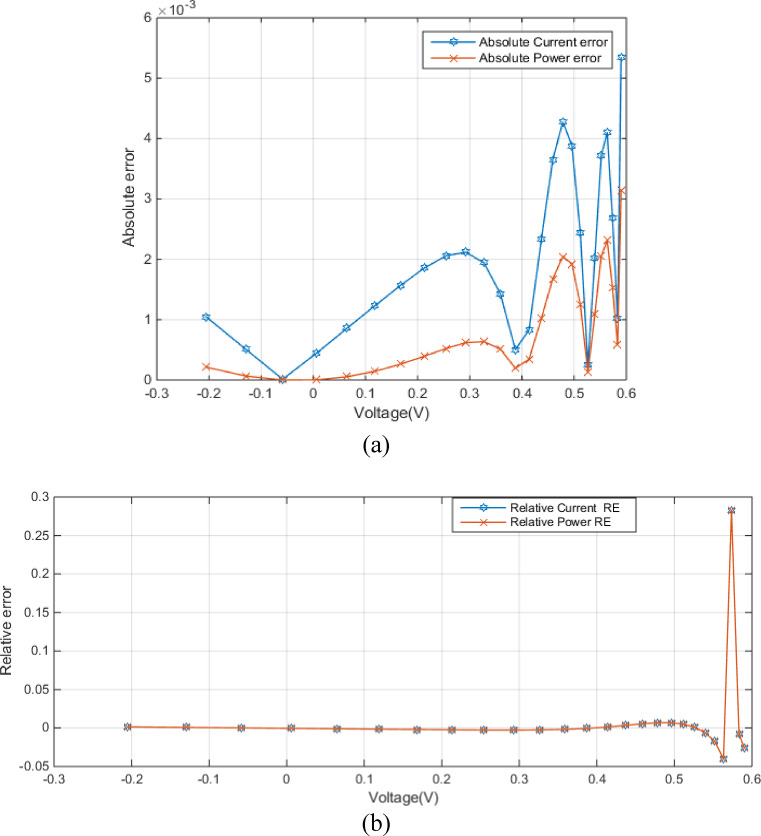
Error-index values for experimental and simulated current data in the DDM (**a**) (IAE) (**b**) (RE).

Figure 7 (a) shows the absolute error between experimental and simulated current and power data in the DDM as a function of voltage. Figure 7 (a) shows relatively low errors between experimental and simulated data, especially at lower voltages, which implies MRQIO’s accurate parameter extraction. Besides, as shown in Fig. 7 (b) the near-zero relative errors across most of the voltage range demonstrate that DDM, when its parameters are extracted using MRQIO, provides a highly accurate representation of the PV module’s behavior under typical operating conditions. This analysis further strengthens the conclusion that MRQIO is a highly effective optimization algorithm for PV parameter extraction.

**Fig. 8:**
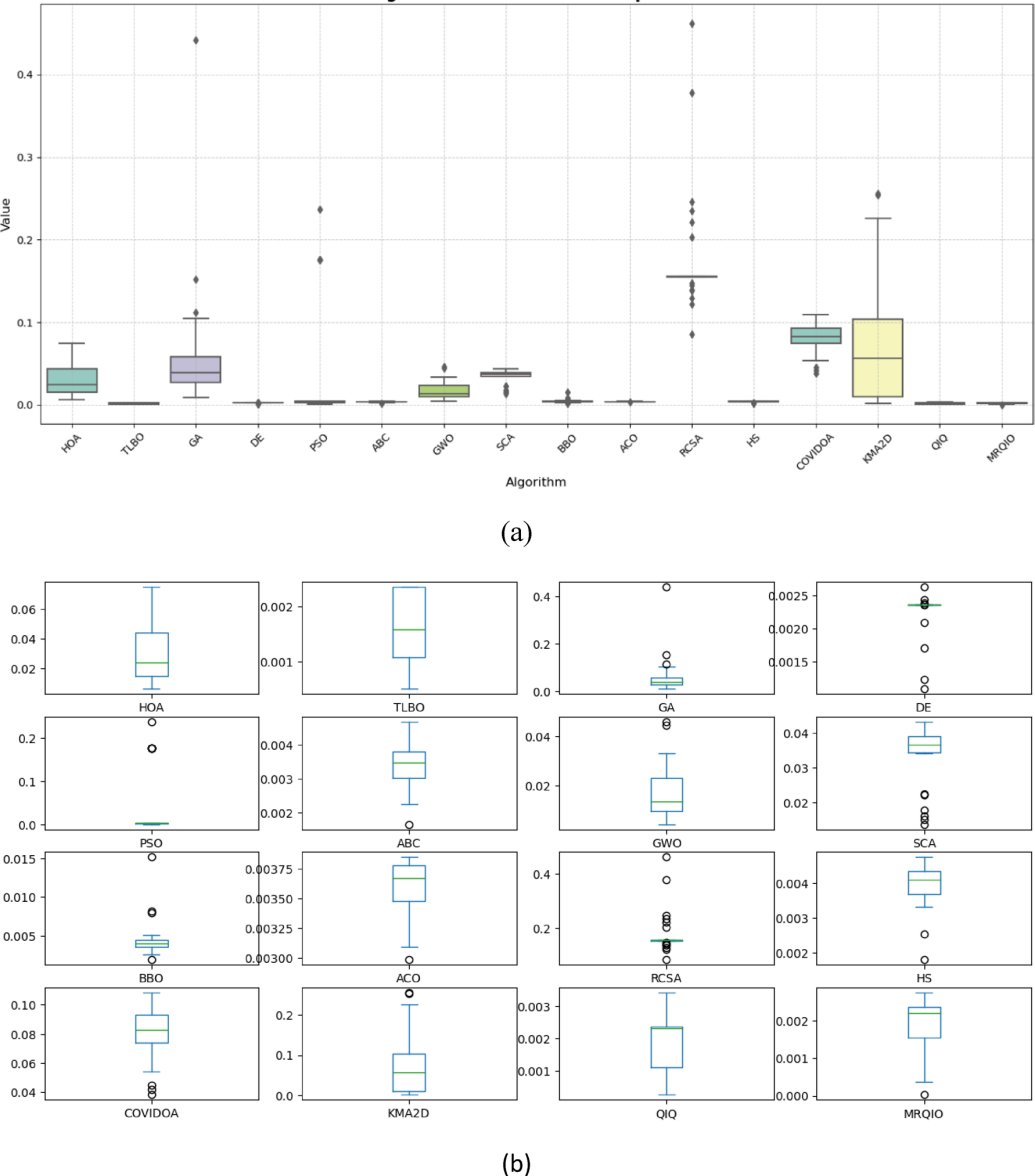
Algorithm Performance Comparison Using RMSE.

Figure 8 provides a comparative analysis of algorithm performance using RMSE metric across different workloads. The figure highlights distinct performance characteristics among the evaluated algorithms, with MRQIQ demonstrating superior efficiency in both convergence speed and solution accuracy. This is particularly evident in its ability to maintain lower error rates and higher consistency during parameter evaluation, outperforming other optimization techniques. The visual comparison underscores MRQIQ’s robustness in handling complex, multi-modal problems, such as PV parameter extraction, where balancing exploration and exploitation is critical.

The results align with the quantitative findings in Tables 6 and 7, where MٌَRQIQ achieves the lowest RMSE values and exhibits stable convergence behavior.

Its performance in STAR-40/24 module testing further validates its applicability to real-world engineering challenges, where precision and computational efficiency are paramount. The divergence in performance trends across workloads also suggests that MRQIQ’s adaptive mechanisms, such as its reinforcement learning and memory-based strategies, effectively navigate diverse search spaces. This reinforces the algorithm’s versatility and positions it as a promising tool for optimizing complex systems beyond photovoltaic modeling.

### Experimental series 3: STM6-40/36 monocrystalline PV module

Table 8 presents a comparison between measured and calculated electrical parameters of the STM6-40/36 PV module using MRQIO. The Integrated Absolute Error for current remained consistently low across all measurements, demonstrating the algorithm’s accuracy in predicting current behavior. The consistently low IAE values for current indicate high accuracy in modeling current behavior. The overall closeness between calculated and measured values suggests that MRQIO provides a reasonable estimation of power output.

**Table 8:** Comparison of calculated and measured values for the STM6-40/36 module.

S.no	$\:{I}_{m}$	$\:{I}_{e}$	IAE(Current)	V	$\:{P}_{m}$	$\:{P}_{e}$	IAE (power)
1	1.663	1.6629	0.000136786	0	0	0	0
2	1.663	1.6627	0.000335912	0.118	0.196234	0.1962	3.96376E-05
3	1.661	1.6591	0.001913431	2.237	3.715657	3.7114	0.004280345
4	1.653	1.6536	0.000648499	5.434	8.982402	8.9859	0.003523944
5	1.65	1.6504	0.000430003	7.26	11.979	11.9821	0.003121821
6	1.645	1.6455	0.000533233	9.68	15.9236	15.9288	0.005161691
7	1.64	1.6396	0.000356997	11.59	19.0076	19.0035	0.004137591
8	1.636	1.6343	0.001657556	12.6	20.6136	20.5927	0.020885202
9	1.629	1.6281	0.000900526	13.37	21.77973	21.7677	0.012040032
10	1.619	1.6193	0.000279425	14.09	22.81171	22.8156	0.003937097
11	1.597	1.6042	0.007154169	14.88	23.76336	23.8698	0.106454036
12	1.581	1.5825	0.001478817	15.59	24.64779	24.6708	0.023054754
13	1.542	1.5426	0.000612417	16.4	25.2888	25.2988	0.01004364
14	1.524	1.521	0.00297429	16.71	25.46604	25.4163	0.049700388
15	1.5	1.4986	0.001365355	16.98	25.47	25.4468	0.023183729
16	1.485	1.4845	0.000524669	17.13	25.43805	25.4291	0.008987574
17	1.465	1.4645	0.000485038	17.32	25.3738	25.3654	0.008400856
18	1.388	1.3854	0.002587946	17.91	24.85908	24.8127	0.046350116
19	1.118	1.1205	0.002504915	19.08	21.33144	21.3792	0.047793774
20	0	−0.0002	0.000163821	21.2	0	−0.0035	0.003472997

Table 9 demonstrates the superior performance of the MRQIO algorithm for parameter extraction of the STM6-40/36 PV module. MRQIO achieved the lowest RMSE (6.77942E-05) among all tested algorithms, indicating the highest accuracy in fitting the model to the measured data.

Furthermore, its low standard deviation (0.002100457) confirms its robustness and consistency in converging to optimal solutions. Compared to other well-established optimization techniques such as GA, PSO, and ABC, MRQIO, along with TLBO and QIO, offers a significant improvement in accuracy and reliability for PV parameter extraction.

**Table 9:** A comparative analysis of different algorithms applied for the STM6-40/36 module.

Algorithms	RMSE			$\:{I}_{ph}$	$\:{I}_{sd}$	$\:{R}_{s}$	$\:{R}_{sh}$	$\:n$
	Std	Mean	Minimum					
HOA	0.02690	0.02652	0.002865	1.66473	1.69350	0.30332	649.07317	55.08335
TLBO	0.00270	0.00045	0.002053	1.66448	0.95793	0.36000	565.99873	52.90560
GA	0.53515	0.53228	0.011878	1.65514	2.33989	0.18592	493.32765	56.45788
DE	0.00378	0.00060	0.002296	1.66370	1.25862	0.33207	622.37358	53.92596
PSO	0.02690	0.06067	0.004979	1.66190	5.26969	0.14772	1000.00000	60.00000
ABC	0.00593	0.00119	0.003594	1.65725	1.14140	0.34231	837.67698	53.53985
GWO	0.02392	0.01678	0.00527	1.66374	5.25497	0.14126	796.02639	59.94413
SCA	0.11586	0.13945	0.013259	1.64566	2.98468	0.13007	1000.00000	57.39485
BBO	0.00618	0.00460	0.002098	1.66501	0.91231	0.36000	538.72395	52.72845
ACO	0.00444	0.00064	0.002114	1.66500	0.90457	0.36000	536.84872	52.69731
RC	2.15152	3.13292	0.195133	1.69207	4.69570	0.16919	977.09507	57.55208
HS	0.00421	0.00134	0.002441	1.66345	1.40877	0.32037	651.21740	54.35798
COVIDOA	0.05814	0.02141	0.02732	1.69530	5.37400	0.36000	933.58079	60.00000
KMA2D	0.26042	0.42067	0.003906	1.65829	1.79423	0.27652	892.67718	55.29997
QIO	0.00212	0.00010	0.008906	1.66451	0.95801	0.36000	565.54458	52.90597
MRQIO	0.00210	0.00007	0.002053	1.66387	0.99056	0.35955	592.95527	53.02740

**Fig. 9:**
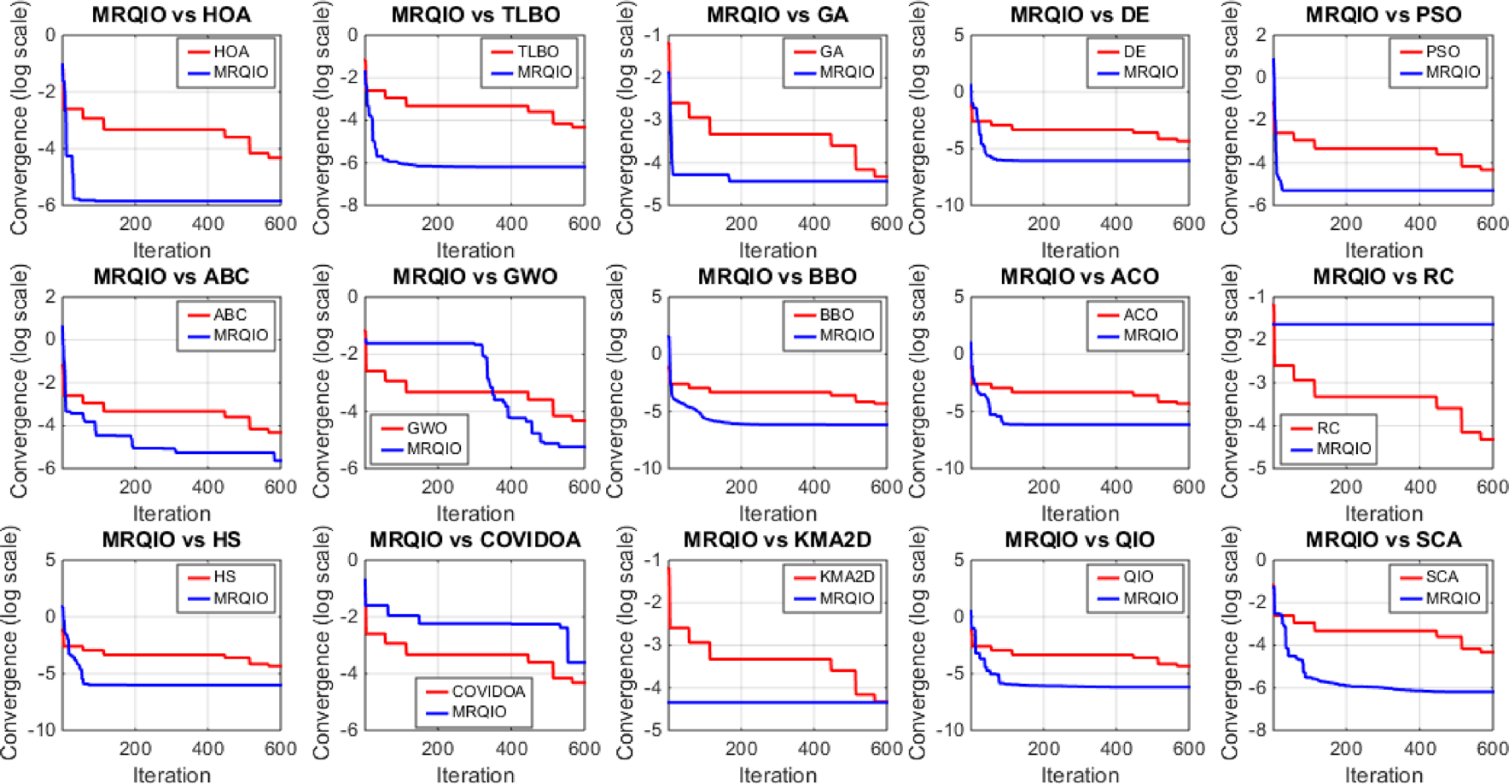
Convergence behavior of MRQIO compared with other optimization algorithms for STM6-40/36 module.

Figure 9 presents a comprehensive comparison of algorithm performance using RMSE metrics across multiple photovoltaic models. The boxplot visualization effectively demonstrates the statistical distribution of results obtained by various optimization algorithms, including the proposed MRQIO method. The figure clearly shows that MRQIO achieves consistently lower median RMSE values with minimal variation compared to competing algorithms, indicating both superior accuracy and remarkable stability in parameter estimation.

The visual representation reveals distinct performance tiers among the evaluated algorithms. While classical methods like GA, PSO, and DE exhibit wider interquartile ranges and higher median errors, MRQIO maintains a compact distribution near the optimal range. This performance consistency across multiple runs underscores the robustness of the reinforcement learning and memory-based mechanisms in handling the multimodal nature of photovoltaic parameter estimation problems. The minimal fluctuation in MRQIO’s results particularly highlights its advantage in practical applications where reliable and repeatable performance is crucial for system design and optimization.

**Fig. 10:**
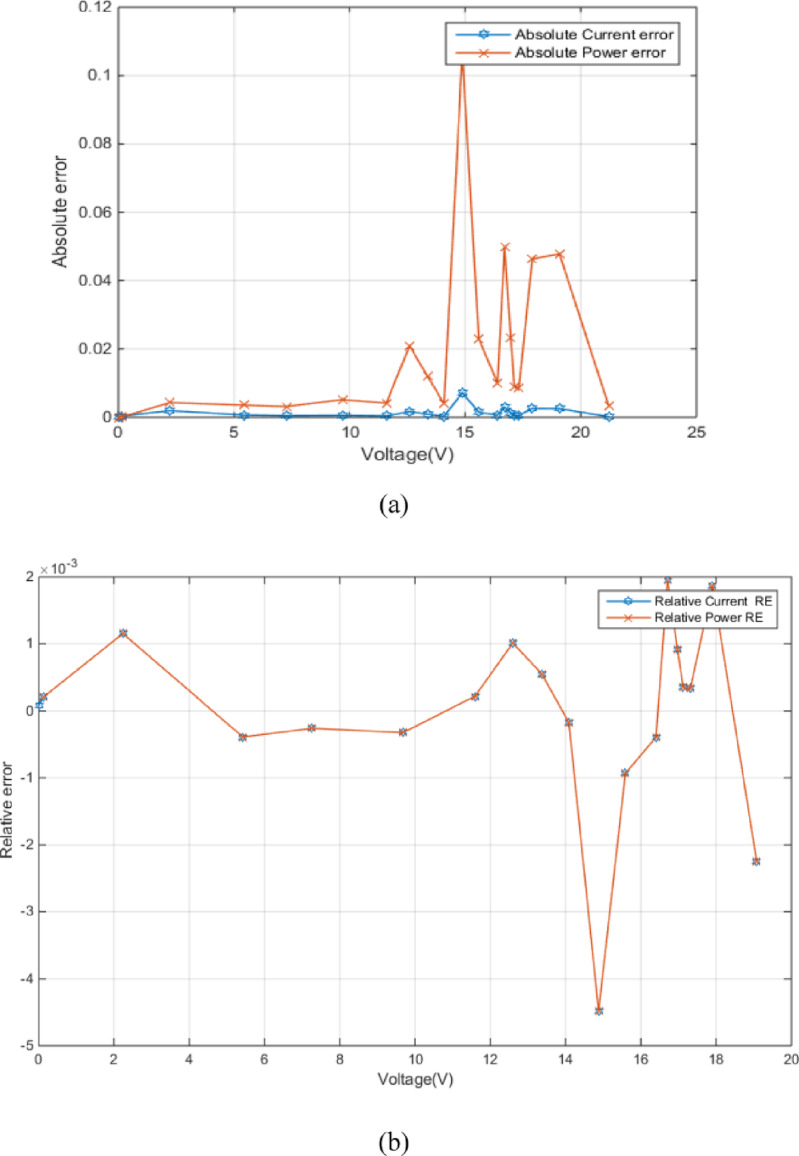
Error-index values for experimental and simulated current data for STM6-40/36 module (**a**) (IAE) (**b**) (RE).

Figure 10 (a) shows absolute errors in current and power as a function of voltage. The absolute current error remains consistently low across the entire voltage range, indicating a good closeness between simulated and experimental current values. Figure 10 (b) presents the relative errors in current and power as a function of voltage. The relative current error remains very low across the entire voltage range, confirming the model’s accuracy in predicting current.

**Fig. 11:**
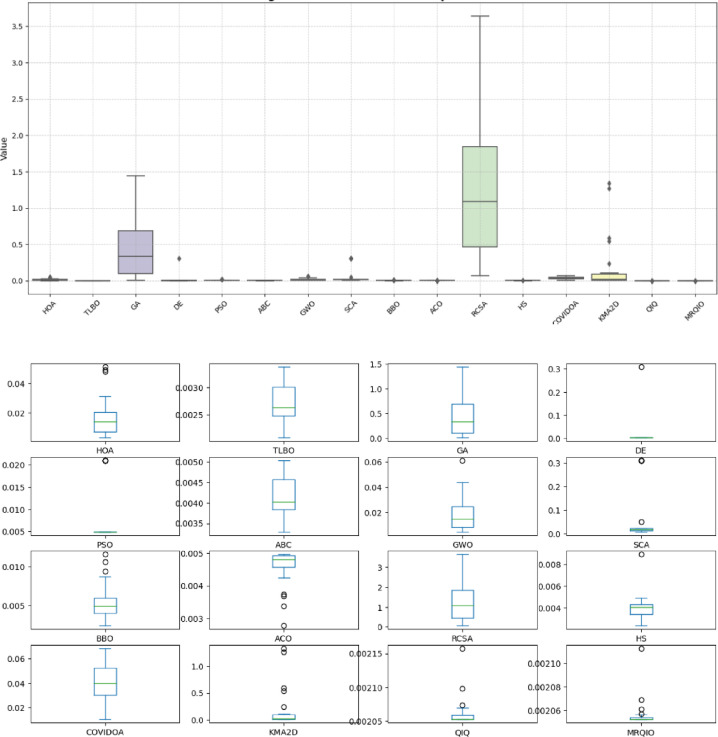
Algorithm Performance Comparison Using RMSE.

Figure 11 provides a compelling visual comparison of algorithm performance using RMSE, demonstrating MRQIO’s consistent superiority across multiple optimizations runs. The boxplot visualization clearly illustrates that MRQIO maintains not only the lowest median error value but also exhibits the most compact interquartile range among all competing algorithms. This combination of optimal accuracy and minimal performance variation underscores the algorithm’s robustness and reliability in handling the complex parameter space of photovoltaic system identification.

The statistical distribution revealed in Fig. 11 highlights MRQIO’s significant advantage over other optimization methods. While several algorithms show wide fluctuations and outlier results, indicating sensitivity to initial conditions or susceptibility to local optima, MRQIO’s consistently narrow error distribution reflects the effectiveness of its reinforcement learning and memory-based mechanisms. This stability is particularly valuable for practical engineering applications where reproducible results are essential, confirming that MRQIO delivers both precision and reliability that surpasses current state-of-the-art optimization techniques for photovoltaic parameter estimation.

### Experimental series 4: STP6-120/36 module

Table 10 presents a comparison between calculated and measured electrical characteristics of the STP6-120/36 PV module, evaluated using the MRQIO optimization algorithm. Generally, the calculated values demonstrate good closeness with the measured data, as evidenced by the relatively low IAE values for both current and power across most data points. Overall, the MRQIO algorithm shows promising performance in accurately predicting the electrical behavior of the STP6-120/36 module under varying operating conditions, with further refinement potentially needed to improve accuracy at low irradiance.

**Table 10:** Comparison of calculated and measured values for the STP6-120/36 module.

S.no	$\:{I}_{m}$	$\:{I}_{e}$	IAE(Current)	V	$\:{P}_{m}$	$\:{P}_{e}$	IAE (power)
1	7.48	7.4693	0.010667585	0	0	0	0
2	7.45	7.4526	0.002551592	9.06	67.497	67.5201	0.023117427
3	7.42	7.4495	0.029455015	9.47	70.2674	70.5463	0.27893899
4	7.44	7.4393	0.000748866	10.32	76.7808	76.7731	0.007728298
5	7.41	7.4205	0.010528673	11.17	82.7697	82.8873	0.117605279
6	7.38	7.3962	0.016212207	11.81	87.1578	87.3493	0.191466159
7	7.37	7.3634	0.006638911	12.36	91.0932	91.0111	0.082056944
8	7.34	7.3315	0.008521654	12.74	93.5116	93.403	0.108565867
9	7.29	7.2841	0.005937992	13.16	95.9364	95.8583	0.078143978
10	7.23	7.2174	0.012645157	13.59	98.2557	98.0839	0.171847685
11	7.1	7.0874	0.012602114	14.17	100.607	100.4284	0.178571958
12	6.97	6.9574	0.012574715	14.58	101.6226	101.4393	0.183339348
13	6.83	6.8132	0.016751258	14.93	101.9719	101.7218	0.250096277
14	6.58	6.5661	0.013875567	15.39	101.2662	101.0527	0.21354497
15	6.36	6.3467	0.013304566	15.71	99.9156	99.7066	0.209014725
16	6	6.0439	0.043922804	16.08	96.48	97.1863	0.706278686
17	5.75	5.7823	0.032291418	16.34	93.955	94.4826	0.527641776
18	5.27	5.2748	0.004752955	16.76	88.3252	88.4049	0.079659521
19	5.07	5.0856	0.015590383	16.9	85.683	85.9465	0.263477469
20	4.79	4.7846	0.005422643	17.1	81.909	81.8163	0.092727192
21	4.56	4.5415	0.018544638	17.25	78.66	78.3401	0.319895
22	4.29	4.2678	0.022226541	17.41	74.6889	74.3019	0.386964075
23	3.83	3.8339	0.003924144	17.65	67.5995	67.6688	0.069261134
24	0	0.0018	0.001808679	19.21	0	0.0347	0.034744727

**Table 11:** A comparative analysis of different algorithms applied for STP6-120/36 module.

Algorithms	RMSE			$\:{I}_{ph}$	$\:{I}_{sd}$	$\:{R}_{s}$	$\:{R}_{sh}$	$\:n$
	Std	Mean	Minimum					
HOA	0.32200	0.37600	0.02179	7.49398	1.98699	0.17106	547.16910	44.88573
TLBO	0.01720	0.00030	0.01683	7.46738	2.43822	0.16485	1453.20190	45.49278
GA	2.84410	2.06360	0.07774	7.37428	1.55573	0.15623	993.56713	44.11085
DE	0.06880	0.25370	0.01679	7.46916	2.38246	0.16515	1050.11734	45.42379
PSO	0.07770	0.09550	0.02207	7.49436	5.23633	0.14964	746.20156	47.93326
ABC	0.03960	0.01040	0.02500	7.45823	4.01064	0.15377	1500.00000	47.07687
GWO	0.12390	0.08640	0.02917	7.49133	9.10516	0.13739	1485.44472	49.86191
SCA	0.36090	0.37100	0.09844	7.41926	9.16425	0.14183	385.51574	50.00000
BBO	0.08140	0.17490	0.01681	7.47637	2.18321	0.16639	584.75172	45.16317
ACO	0.02630	0.00280	0.01811	7.47542	3.63849	0.15742	1500.00000	46.74041
RC	3.76310	6.94750	1.14982	7.05999	7.95629	0.25734	111.23711	49.55510
HS	0.02200	0.00370	0.01683	7.46951	2.18963	0.16670	904.04469	45.17057
COVIDOA	0.39920	0.11560	0.10130	7.65135	9.27551	0.16026	354.37973	50.00000
KMA2D	2.69820	2.39180	0.02911	7.49882	4.80171	0.15793	1249.39470	47.66825
QIO	1.75820	0.00513	0.80142	7.47159	2.32127	0.16551	831.61165	45.34591
MRQIO	0.01680	0.00001	0.01678	7.47064	2.40111	0.16495	944.53968	45.44754

**Fig. 12:**
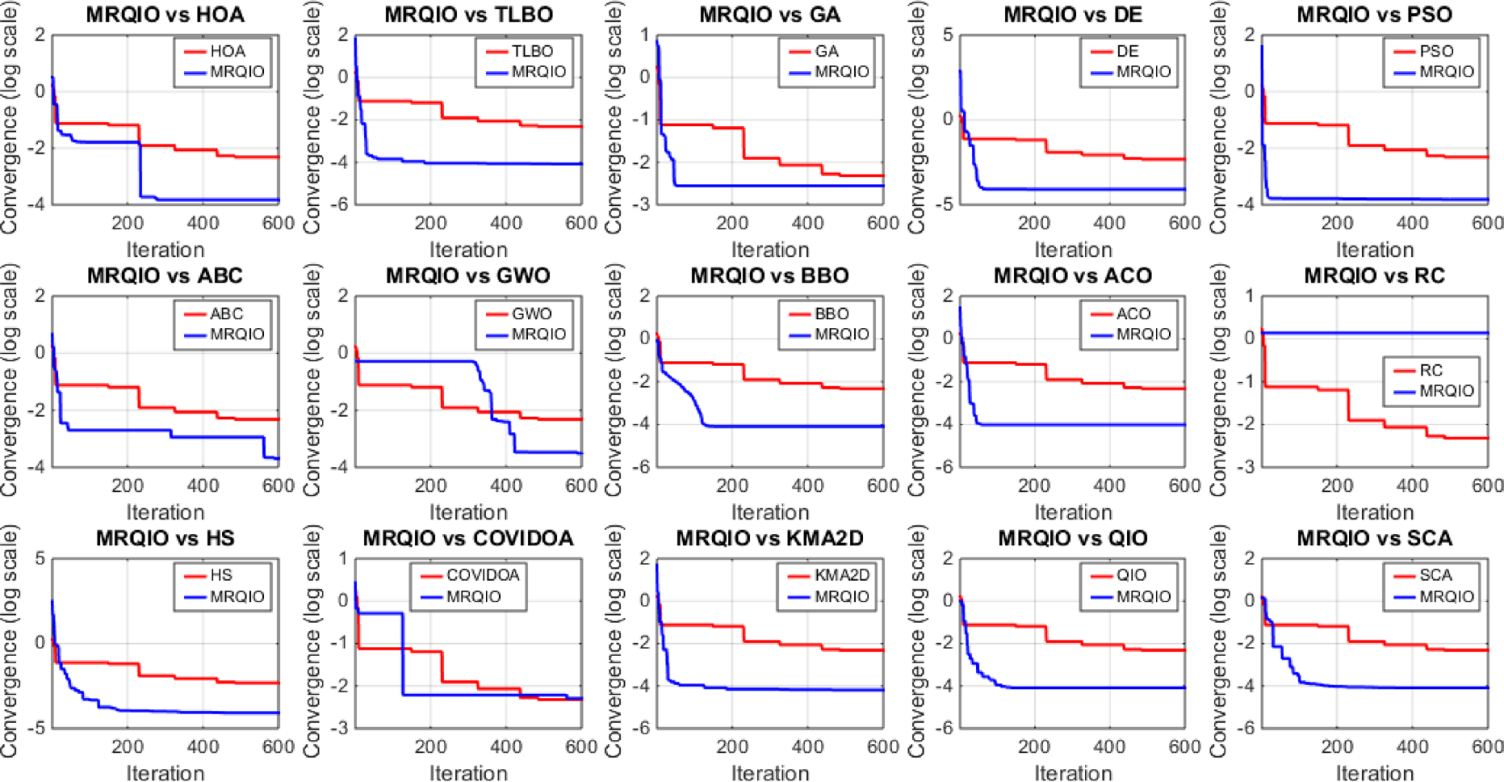
Convergence behavior of MRQIO compared with other optimization algorithms for STP6-120/36 module.

Table 11 provides a comparative analysis of various optimization algorithms applied to model the STP6-120/36 PV module. The table presents key performance metrics, including RMSE with its standard deviation and mean, along with extracted electrical parameters. Notably, the MRQIO algorithm exhibit the lowest RMSE values, suggesting superior performance in accurately characterizing the PV module’s electrical behavior. In contrast, other algorithms demonstrate significantly higher RMSE values, indicating less accurate parameter extraction. The consistent low RMSE and stable parameter extraction of the MRQIO algorithm suggest its robustness and effectiveness in accurately modeling the STP6-120/36 PV module Fig. 12 .

Figure 8 shows illustrate the optimization process of competing algorithms versus MRQIQ by plotting Log (RMSE) against the number of iterations. This visualization effectively demonstrates the rate at which each algorithm approaches the optimal solution. As shown in the graph, all algorithms exhibit a rapid decrease in RMSE during the initial iterations, indicating efficient exploration of the search space. Notably, TLBO, QIO, and MRQIO achieve faster convergence compared to DE, reaching a stable minimum RMSE value within approximately 100 iterations. This suggests that these three algorithms possess superior search capabilities and can more efficiently identify optimal parameter sets. While DE also converges with a similar RMSE value eventually, its convergence rate is slower, requiring more iterations to reach the stable solution. The similar final RMSE values of TLBO, QIO, and MRQIO corroborate the findings in Table 7, further highlighting their effectiveness in accurately modeling the PV module.

**Fig. 13:**
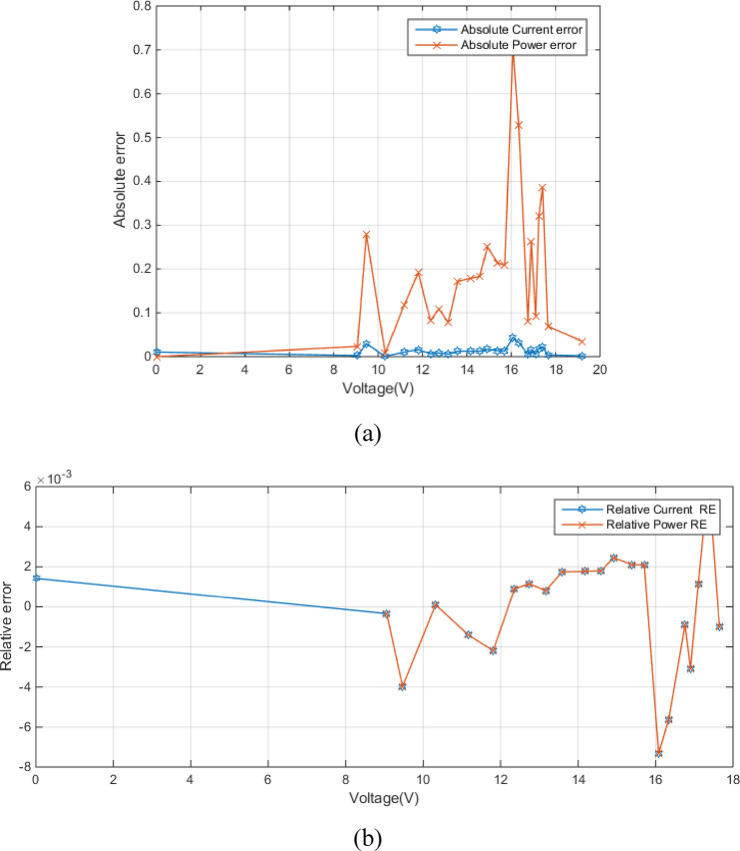
Error-index values for experimental and simulated current data for STP6-120/36 module (**a**) (IAE) (**b**) (RE).

Figure 13 (a) illustrates the error-index values for experimental and simulated current data of the STP6-120/36 module, specifically showing the Absolute Error (AE) for both current and power as a function of voltage. The plot reveals that the absolute current error remains consistently low across the entire voltage range, generally staying below 0.05. This indicates a strong closeness between the simulated and experimental current values.

Figure 13(b) presents the relative error (RE) for both current and power as a function of voltage for the STP6-120/36 module. Unlike the absolute error shown in Fig. 13 (a), the relative error provides a normalized perspective on the discrepancies between simulated and experimental data, expressing the error as a fraction of the measured value. The relative current error remains consistently small, generally within ± 2 × 10⁻³, indicating a high degree of accuracy in current prediction across the voltage range.

**Fig. 14:**
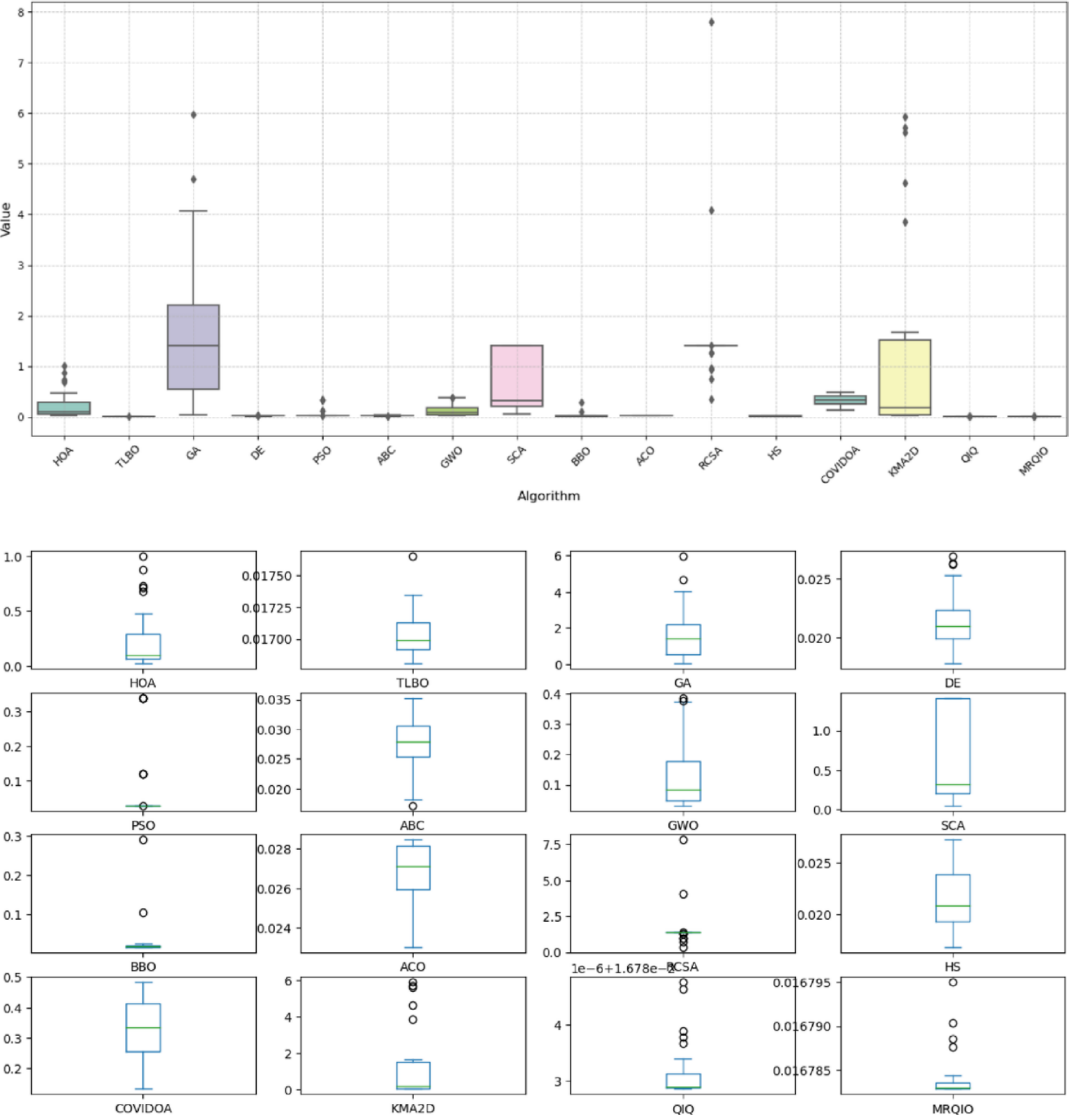
Algorithm performance comparison using RMSE.

Figure 14 shows that MRQIQ achieves highest and consistent median whereas other algorithms such as SCA, GWO, and HOA show lowest performance.

### Experimental series 5: Photowatt-PWP201 module

Table 12 presents a comparison of calculated and measured values for a Photowatt-PWP201 photovoltaic module. For both current and power, the IAE fluctuates across the different data points, indicating varying levels of accuracy under different conditions. Notably, some of the IAE values, particularly for power, are relatively high, suggesting potential areas for model refinement. Further investigation into the conditions corresponding to these higher errors could provide insights into the model’s limitations and guide future improvements. This detailed comparison of calculated and measured values, along with the IAE analysis, provides a valuable assessment of the model’s predictive capabilities for the Photowatt-PWP201 module.

**Table 12:** Comparison of calculated and measured values for the *Photowatt-PWP201 module*.

S.no	$\:{I}_{m}$	$\:{I}_{e}$	IAE(Current)	V	$\:{P}_{m}$	$\:{P}_{e}$	IAE (power)
1	1.031	1.0291	0.002363189	0.1248	0.1287	0.1284	0.000294926
2	1.03	1.0274	0.002607859	1.8093	1.8636	1.8589	0.004718399
3	1.026	1.0257	0.000253057	3.3511	3.4382	3.4374	0.000848018
4	1.022	1.0241	0.002107082	4.7622	4.867	4.877	0.010034345
5	1.018	1.0223	0.004287348	6.0538	6.1628	6.1887	0.025954745
6	1.015	1.0199	0.0044229	7.2364	7.3486	7.3806	0.032005876
7	1.014	1.0164	0.002353388	8.3189	8.4354	8.4549	0.019577601
8	1.01	1.0105	0.000486235	9.3097	9.4028	9.4073	0.004526702
9	1.003	1.0006	0.002879192	10.216	10.252	10.222	0.029414691
10	0.988	0.9845	0.003456181	11.044	10.912	10.874	0.038173178
11	0.963	0.9595	0.003478036	11.801	11.365	11.324	0.041047084
12	0.925	0.9228	0.002655877	12.492	11.562	11.529	0.033179605
13	0.872	0.8726	0.000108897	13.123	11.449	11.451	0.001429067
14	0.807	0.8073	0.000214874	13.698	11.061	11.058	0.002943402
15	0.726	0.7283	0.001846445	14.222	10.332	10.358	0.026260322
16	0.634	0.6371	0.002644714	14.699	9.3268	9.3657	0.038875967
17	0.534	0.5362	0.001714961	15.134	8.0894	8.1154	0.02595525
18	0.427	0.4295	0.002008398	15.531	6.6395	6.6707	0.031192626
19	0.318	0.3188	0.00026737	15.892	5.0619	5.0661	0.004249287
20	0.208	0.2074	0.001119892	16.222	3.3825	3.3643	0.018167897
21	0.101	0.0962	0.004842607	16.524	1.6689	1.5889	0.080019727
22	−0.008	−0.0083	0.000331005	16.798	−0.1344	−0.14	0.00556046
23	−0.111	−0.1109	6.38823E-05	17.049	−1.8925	−1.8914	0.001089187
24	−0.209	−0.2092	0.000238549	17.279	−3.6114	−3.6155	0.004121967
25	−0.303	−0.3008	0.002156389	17.488	−5.299	−5.2613	0.037712002

**Table 13:** A comparative analysis of different algorithms applied for *Photowatt-PWP201 module*.

Algorithms	RMSE			$\:{I}_{ph}$	$\:{I}_{sd}$	$\:{R}_{s}$	$\:{R}_{sh}$	$\:n$
	Std	Mean	Minimum					
HOA	0.00250	0.00003	0.00292	1.03019	3.55026	1.19942	1033.27696	48.71547
TLBO	0.27080	0.18270	0.00243	1.03569	1.32844	1.29674	614.58075	45.17557
GA	0.03830	0.10720	0.00523	1.03051	3.48226	1.20127	981.98226	48.64283
DE	0.05210	0.10400	0.00243	1.03040	3.44645	1.20340	1000.92654	48.60184
PSO	0.00740	0.00250	0.00243	1.02779	4.92165	1.15635	1571.27591	50.00000
ABC	0.02480	0.01790	0.00297	1.02364	4.90348	1.14589	1259.45993	49.97668
GWO	0.14850	0.10680	0.00312	1.05458	4.61854	0.99534	249.45496	50.00000
SCA	0.00510	0.00390	0.02067	1.03059	3.38729	1.20431	961.37873	48.53691
BBO	0.00260	0.00020	0.00243	1.02999	3.59252	1.19846	1053.08300	48.76141
ACO	2.13460	1.71150	0.00243	1.06319	5.80206	0.71190	1041.03278	49.62903
RC	0.00350	0.00170	0.17693	1.03053	3.41301	1.20393	975.27038	48.56543
HS	0.09660	0.02250	0.00243	1.06873	5.01810	1.29029	1444.02697	50.00000
COVIDOA	0.52260	0.20660	0.03050	0.96237	1.78947	0.83966	1999.88642	46.48462
KMA2D	0.42560	1.76600	0.05126	1.03049	3.49097	1.20104	985.68269	48.65235
QIO	0.03420	0.00400	0.00243	1.03054	3.47155	1.20160	978.19437	48.63106
MRQIO	0.00250	0.00003	0.00243	1.03019	3.55026	1.19942	1033.27696	48.71547

Table 13 presents a comparative analysis of various optimization algorithms applied to a Photowatt-PWP201 photovoltaic module, likely for parameter extraction of a PV model. MRQIO has very low RMSE values, suggesting superior performance in this context. Conversely, other algorithms exhibit significantly higher RMSE, indicating poorer performance for this application.

**Fig. 15:**
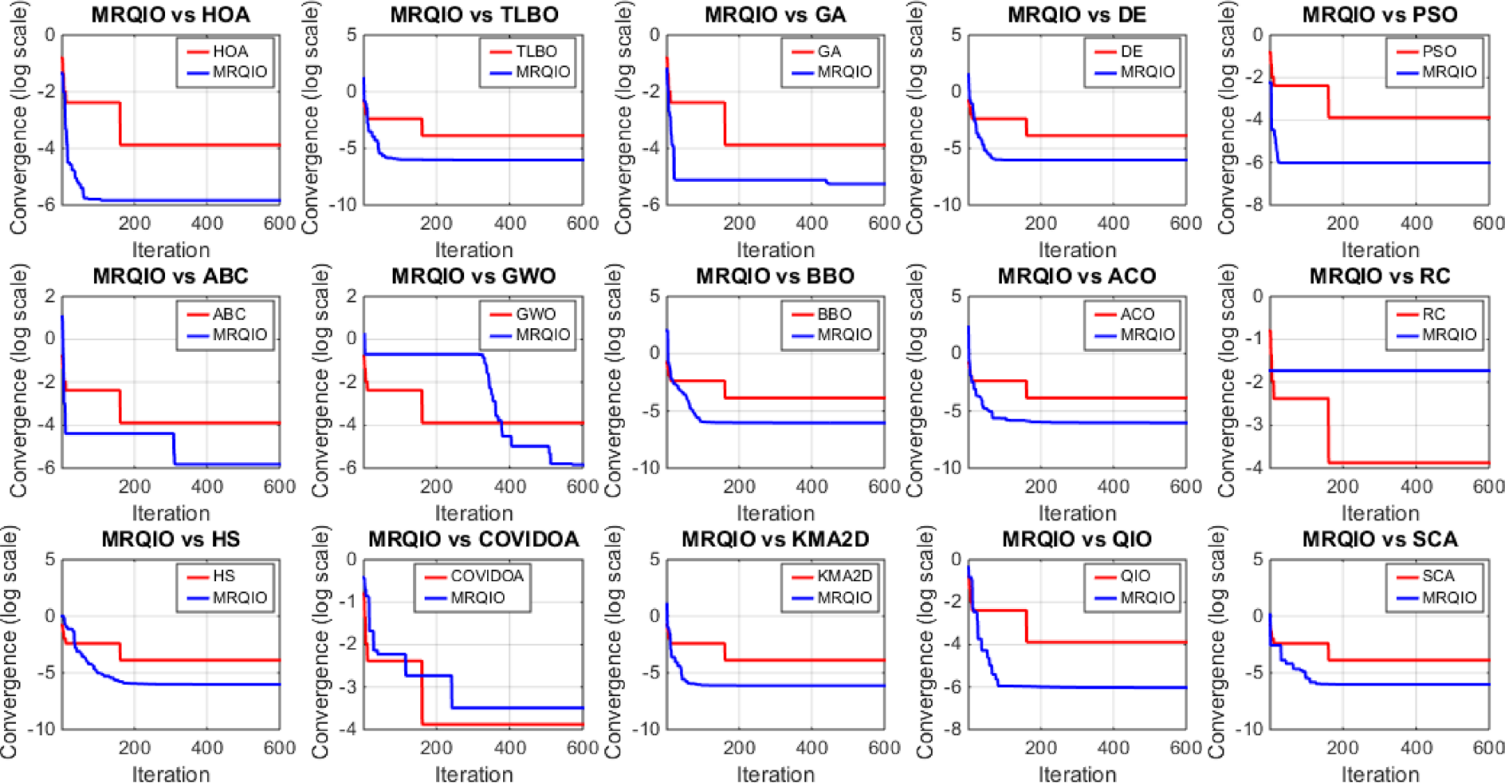
Convergence behavior of MRQIO compared with other optimization algorithms for *Photowatt-PWP201 module*.

**Fig. 16:**
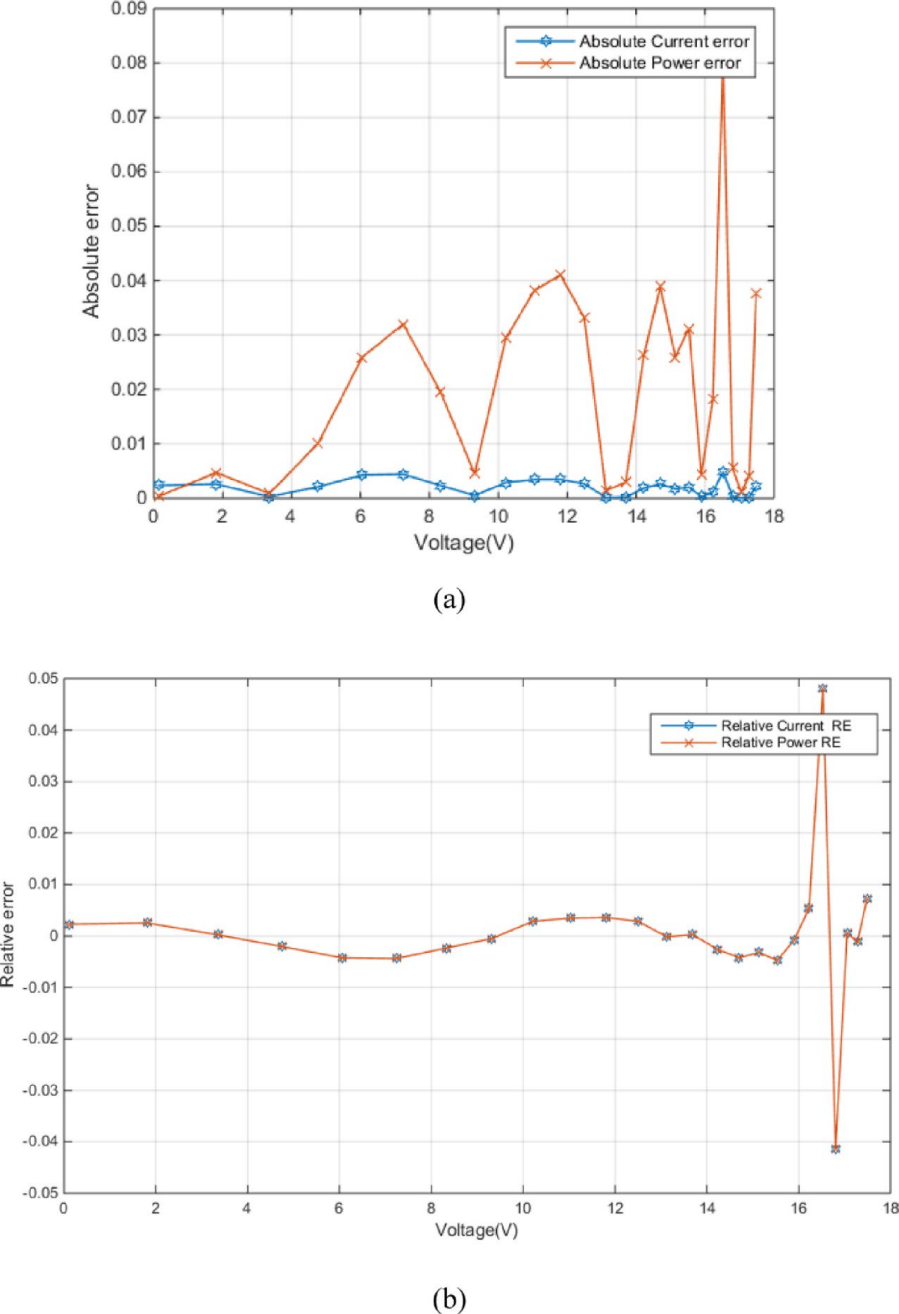
Error-index values for experimental and simulated current data for *Photowatt-PWP201 module* (**a**) (IAE) (**b**) (RE).

Figure 15 visually demonstrates the convergence behavior of MRQIQ versus competing algorithms. MRQIO shows the best performance, achieving faster convergence and a lower final RMSE, making them the preferred choices for parameter extraction of the Photowatt-PWP201 module based on this analysis Fig. 16.

**Fig. 17:**
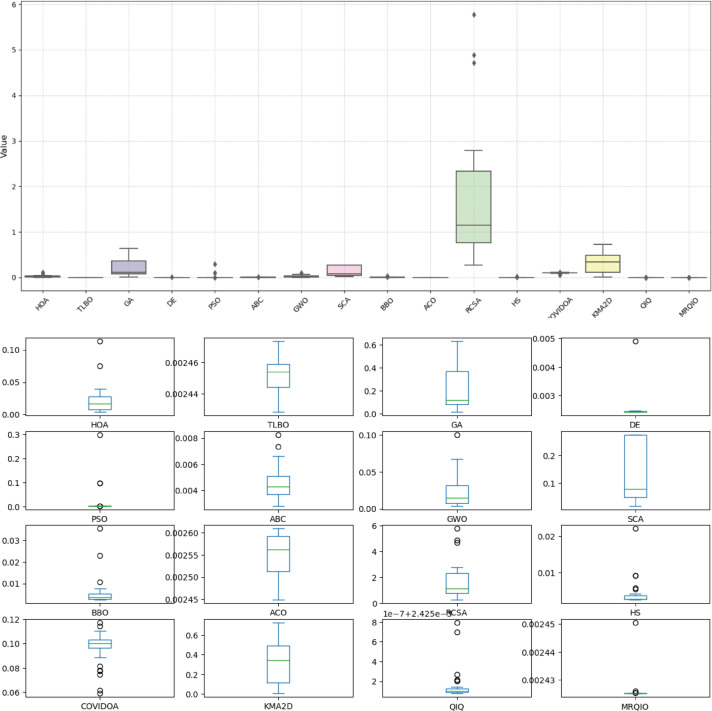
Algorithm performance comparison using RMSE.

Figure 17 shows performance of median value of RMSE for MRQIO against other competing algorithm where MRQIO prove consistent and better performance than other algorithms.

### Wilcoxon test analysis

Table 14 presents the results of Wilcoxon’s rank sum test, comparing the performance of various optimization algorithms across five different PV module models. The values shown are p-values, which quantify the statistical significance of the difference between each algorithm and MRQIO algorithm.

**Table 14:** Results of the wilcoxon’s rank sum test.

Algorithms	RTC SDM	RTC DDM	STP6-120/36	STM6-40/3	PWP201
HOA	0.008544	0.000179	0.00376	0.004279	0.006458
TLBO	2.63E-11	8.96E-16	2.43E-10	9.36E-11	2.01E-08
GA	3.68E-09	8.52E-09	1.52E-14	3.16E-12	6.47E-09
DE	7.57E-07	6.27E-09	0.000335	9.26E-06	1.53E-05
PSO	0.205895	0.99295	0.819873	0.591759	0.330722
ABC	0.965844	0.675933	0.909623	0.646377	0.825167
GWO	0.086613	0.061313	0.041637	0.002597	0.086115
SCA	2.62E-05	0.001464	6.70E-05	1.13E-05	3.62E-06
BBO	0.198679	0.034235	0.000587	0.028278	0.153274
ACO	0.03951	0.000158	0.019129	0.002331	1.75E-05
RC	7.50E-14	5.68E-16	7.81E-15	7.91E-19	1.56E-18
HS	0.0047	0.086863	4.32E-05	0.000257	0.005656
COVIDOA	1.42E-11	5.11E-09	5.96E-07	1.34E-08	2.58E-06
KMA2D	5.78E-13	2.34E-13	9.21E-13	1.27E-06	1.34E-14
QIO	2.19E-17	4.92E-12	3.17E-17	3.33E-17	5.80E-17
MRQIO	4.60E-17	2.04E-12	6.33E-18	2.79E-17	1.61E-17

A p-value below a chosen significance level (commonly 0.05) indicates a statistically significant difference, suggesting that the two algorithms being compared do not perform similarly. Conversely, a p-value above 0.05 suggests no statistically significant difference, implying comparable performance.

Focusing on MRQIO, we observe consistently very low p-values (on the order of 10⁻¹² to 10⁻¹⁸) across all modules. These extremely low p-values indicate a statistically significant difference between MRQIO and the other algorithms it was compared to (presumably all the others listed in the table). This means that MRQIO’s performance is demonstrably different, and in this case, superior to the other algorithms. The consistency of these very low p-values across all module types (RTC SDM, RTC DDM, STP6-120/36, STM6-40/3, and PWP201) further reinforces the conclusion that MRQIO is a robust and effective optimization algorithm for PV module parameter extraction, consistently outperforming the other tested algorithms.

### Friedman test results analysis

Table 15 shows Friedman test results across all photovoltaic models. The results reveal highly significant performance difference among the competing algorithms $\:p-\text{v}\text{a}\text{l}\text{u}\text{e}<{10}^{-72})$. The MRQIQ, QIQ, and TLBO achieved best ranks. In contrast, GA, RCSA, and COVIDOA showed lower performance. The results confirm the robustness and effectiveness of the proposed MRQIQ.

**Table 15:** Friedman test results for various photovoltaic Models.

Model	Top Algorithms (Lowest Ranks)	Worst Algorithms (Highest Ranks)	Friedman Test Statistic	*p*-value	Statistical Decision
SDM	MRQIO (1.47), QIQ (1.67), TLBO (3.10)	COVIDOA (14.40), RCSA (15.67)	406.93	2.42 × 10⁻⁷⁷	Significant differences
DDM	TLBO (2.17), QIQ (2.67), MRQIO (2.83)	COVIDOA (14.23), RCSA (15.67)	382.14	3.90 × 10⁻⁷²	Significant differences
STM6	MRQIO (1.47), QIQ (1.53), TLBO (3.23)	GA (14.10), RCSA (15.73)	395.48	6.17 × 10⁻⁷⁵	Significant differences
STP6	QIQ (1.40), MRQIO (1.60), TLBO (3.13)	GA (14.33), RCSA (14.93)	400.21	6.24 × 10⁻⁷⁶	Significant differences
PWP201	QIQ (1.63), MRQIO (1.77), DE (2.93), TLBO (3.87)	KMA2D (13.80), RCSA (15.93)	418.65	8.29 × 10⁻⁸⁰	Significant differences

### Computational time analysis of optimization algorithms

Table 16 summarize the average and standard deviation of different optimization algorithms. it is observed that the classical algorithms such as GA, DE, and PSO achieve the least running time, often below 0.5 s on average with small deviation which indicating stable and efficient execution. In contrast, more advanced algorithms such as MRQIQ, QIQ, and COVIDOA have higher running times which exceeding 2 s. This behavior indicating high complexity of these algorithms which integrate adaptive mechanism for exploration and exploitation. Although, MRQIQ requires greater computational time, its superior convergence performance justifies the additional time cost which provide a trade-off between solution quality and computational time.

**Table 16:** AVERAGE and standard deviation of computation times for the evaluated algorithms.

	Metric	RTC SDM	RTC DDM	STP6-120/36	STM6-40/36	PWP201
HOA	Avg.	0.422	0.467	0.512	0.447	0.438
	Std	0.038	0.059	0.336	0.060	0.049
TLBO	Avg.	1.038	1.124	1.140	1.153	1.106
	Std	0.081	0.062	0.228	0.175	0.140
GA	Avg.	0.359	0.408	0.381	0.401	0.395
	Std	0.021	0.036	0.056	0.100	0.071
DE	Avg.	0.459	0.488	0.494	0.506	0.483
	Std	0.023	0.036	0.061	0.104	0.066
PSO	Avg.	0.273	0.308	0.278	0.288	0.285
	Std	0.017	0.020	0.035	0.076	0.035
ABC	Avg.	1.024	1.148	1.150	1.135	1.107
	Std	0.049	0.172	0.172	0.151	0.104
GWO	Avg.	0.316	0.365	0.336	0.333	0.330
	Std	0.043	0.043	0.052	0.050	0.034
SCA	Avg.	0.292	0.331	0.302	0.310	0.303
	Std	0.024	0.032	0.037	0.062	0.041
BBO	Avg.	1.211	1.592	1.302	1.452	1.409
	Std	0.092	0.091	0.135	0.336	0.485
ACO	Avg.	1.437	1.809	1.490	1.607	1.666
	Std	0.115	0.091	0.172	0.328	0.478
RCSA	Avg.	48.398	51.366	50.280	51.466	52.118
	Std	3.896	1.125	3.842	4.029	6.836
HS	Avg.	0.994	1.214	1.078	1.102	1.108
	Std	0.071	0.071	0.123	0.168	0.237
COVIDOA	Avg.	2.060	2.138	2.136	2.178	2.176
	Std	0.141	0.097	0.263	0.284	0.398
KMA2D	Avg.	1.667	1.816	1.763	1.782	1.790
	Std	0.109	0.074	0.163	0.167	0.258
QIO	Avg.	2.187	2.780	2.357	2.353	2.520
	Std	0.165	0.097	0.234	0.179	0.602
MRQIQ	Avg.	2.186	2.793	2.398	2.433	2.475
	Std	0.191	0.135	0.495	0.252	0.528

Based on the experimental results, it can be observed that MRQIQI shows a superior convergence, accuracy, and robustness compared to state-of-the-art method due to reinforcement learning that balance between exploration and exploitation, memory-based diversity enhancement, and interpolation-based local research. However, MRQIQ still has potential weaknesses including higher computational demands and careful adaptation for real-world applications.

## Conclusions

### Outcomes

This study successfully developed the MRQIO algorithm, an enhanced version of QIO that effectively mitigates its tendency for premature convergence through the synergistic integration of reinforcement learning and a memory-based mechanism. The key outcome is the demonstration of MRQIO’s superior accuracy and robustness in estimating parameters for various PV models. The algorithm consistently achieved the lowest RMSE values across all tested cases, outperforming a wide range of classical and advanced meta-heuristics. This performance is attributed to its self-adaptive ability to balance global exploration and local refinement, leading to faster convergence and a higher likelihood of locating the global optimum in complex, multimodal search spaces.

### Limitations

Despite its superior performance, the proposed MRQIO algorithm has certain limitations. The primary drawback is its higher computational cost compared to simpler algorithms like PSO, GA, and DE, due to the overhead of the reinforcement learning component and memory management. This could be a constraint on applications requiring real-time or ultra-fast parameter estimation. Additionally, while the algorithm was tested on standard PV models, its performance in the presence of significant measurement noise or under rapidly changing real-world environmental conditions requires further investigation.

### Future work

Future research will focus on several avenues. First, efforts will be made to optimize the computational efficiency of MRQIO, potentially through code optimization or simplified RL policies, to make it more suitable for time-sensitive applications. Second, the algorithm’s applicability will be extended to other complex problems in renewable energy, such as parameter estimation for fuel cells and wind turbine systems, and the optimization of hybrid energy microgrids. Finally, exploring the integration of MRQIO with deep learning models for real-time system diagnostics and prognostics presents a promising direction for intelligent energy management systems.

## Data Availability

All data generated or analyzed during this study are included in this published article.
